# HDOMO: Smart Sensor Integration for an Active and Independent Longevity of the Elderly

**DOI:** 10.3390/s17112610

**Published:** 2017-11-13

**Authors:** Emanuele Frontoni, Rama Pollini, Paola Russo, Primo Zingaretti, Graziano Cerri

**Affiliations:** Department of Information Engineering, Università Politecnica delle Marche, I-60131 Ancona, Italy; r.pollini@pm.univpm.it (R.P.); p.russo@univpm.it (P.R.); p.zingaretti@univpm.it (P.Z.); g.cerri@univpm.it (G.C.)

**Keywords:** smart home, smart objects, ambient intelligent, interoperability, elderly, AAL, intelligent systems

## Abstract

The aim of this paper is to present the main results of HDOMO, an Ambient Assisted Living (AAL) project that involved 16 Small and Medium Enterprises (SMEs) and 2 research institutes. The objective of the project was to create an autonomous and automated domestic environment, primarily for elderly people and people with physical and motor disabilities. A known and familiar environment should help users in their daily activities and it should act as a virtual caregiver by calling, if necessary, relief efforts. Substantially, the aim of the project is to simplify the life of people in need of support, while keeping them autonomous in their private environment. From a technical point of view, the project provides the use of different Smart Objects (SOs), able to communicate among each other, in a cloud base infrastructure, and with the assisted users and their caregivers, in a perspective of interoperability and standardization of devices, usability and effectiveness of alarm systems. In the state of the art there are projects that achieve only a few of the elements listed. The HDOMO project aims to achieve all of them in one single project effectively. The experimental trials performed in a real scenario demonstrated the accuracy and efficiency of the system in extracting and processing data in real time to promptly acting, and in providing timely response to the needs of the user by integrating and confirming main alarms with different interoperable smart sensors. The article proposes a new technique to improve the accuracy of the system in detecting alarms using a multi-SO approach with information fusion between different devices, proving that this architecture can provide robust and reliable results on real environments.

## 1. Introduction

### 1.1. Main Challenges of AAL

In recent years, particularly in the industrialized countries, society has been moving towards an important demographic change also known as the ageing society. This change is due to improve life expectancy, which causes the ageing of population. In order to contain the expenses for the health care within the limits of economic possibility, it is necessary to find productive and innovative solutions [1,2]. According to the World Health Organization [3], the number of elderly people (people of 60 years of age and older) in the world is about 650 millions and by 2050 it will reach 2 billions. European population will keep on growing older a well. In 2008 the population over the age of 65 was over 17%, and in 2060 it will rise to 30%, while, the population over the age of 85 will go from 4% to 12% [4]. Considering this trend, it is important to implement smart solutions for elderly care since they should remain independent and able to work for a longer time. This can be achieved with technology. For this purpose, Ambient Assisted Living (AAL) applications have attracted a growing attention in the scientific community since they involve emerging and innovative technological solutions, providing embedded systems in the home environment. The aim of AAL is to improve the quality of life, to reduce costs for independent living [5,6,7,8], to increase the self-confidence and the autonomy of elderly or ill people while enhancing their security.

### 1.2. Main Objectives of HDOMO Project

In this context, we propose the complex HDOMO (Human Based DOmotics) project, which aims to improve the life of older people and/or people with disabilities by ensuring their independence in their familiar, domestic environment. The home becomes smart, interacting with people and executing commands given but it is also able, if necessary, to act independently, in case of emergency (for example a person falls on the floor or when the presence of smoke is detected). A series of actions are immediately triggered to ensure the safety of people and a timely arrival of relief efforts. An important aspect to highlight is that home automation 2.0 does not impact living conditions in any way. Its first objective was to create highly technological objects of domestic use making them non-invasive and easy to use, to facilitate hard and uneasy tasks and take them much simpler. In their home, older people live better, are more at ease, maintain contact with a known environment, keeping intact their “world”. HDOMO is an Italian project (supported by indirect EU funding in the POR action of Marche region) which involves 16 SMEs and 2 research institutes (http://vrai.dii.univpm.it/aal) and is mainly focused on Human Behaviours Analysis (HBA) in AAL. This project aims to propose an innovative idea of an interoperable embedded intelligent system where a series of low cost smart sensors can analyse human behaviours to obtain interactivity and statistical data, mainly devoted to HBA in intelligent AAL environments. HDOMO defines a framework that can be described and split in modules: a low level indoor smart sensor family (which manages localization, access management, interactions, and gesture analysis), a gateway installed inside AAL environment capable of smart sensor management and low level interoperability, a communication layer sending data to a cloud based web architecture responsible of HBA classification and alarm management. One of the innovations of the system is the use of vision sensors (both RGB and RGBD) for people tracking and interaction analysis, where the depth data has been used to prevent problems with changes in appearence variation and to evaluate users’ activities inside home environment. In addition, the set of interactions are monitored and analysed with the main goal of having a better knowledge of users’ activities, using real data in real time. All information coming from this HBA tool can be used to provide basic data gathered in real time for an AAL environment. This data paired with classical home automation data is used to classify correct and incorrect behaviours using a machine learning approach. In summary, the following objectives of the HDOMO project can be specified:

- The implementation of an innovative and integrated system able to improve the quality of life of older people (both self-sufficient and with frailty and chronicity associated with ageing), enabling them to remain independent in their home, to maintain good health conditions and to have an active role in managing their own health.
- The design and implementation of an open, free and accessible “intelligent framework”, as a supporting structure and enabling tool for various and multifunctional fields (energy, comfort, safety and security management), able to learn the customs of users, to know their capabilities, to monitor their interactions and to infer from and to react to unusual behaviours, enabling alarms or activating procedures of internal checking, which may include user interaction.
- The study and the development of innovative and technological solutions for behaviour analysis, integration with next generation of smart objects (SOs) for localization applications, interaction, analysis of vital parameters, basing much of their analysis of data on the possibility of using audio and video analysis systems.
- The development of methodologies and technologies for advanced Human Machine Interaction, implementing user interfaces that are holistic and adaptive (“User-Centred Design”), accessible by the different types of users (“Design for All”).

In the state of the art there are projects that achieve only a few of the objectives listed. The HDOMO project aims to achieve all of them in one single project effectively.

### 1.3. Background

The integration of solutions among different living environments is mainly functional to necessities of users. It also allows to achieve important synergies in the development of technologies and applications. The development of the project has involved the collaboration of international hi-tech companies, centres of research and end users of the care sector involved in the phases of analysis and experimentation. The concepts of Ambient Intelligence (AmI) [9,10,11] and AAL provide a vision of the information society in which the emphasis is on a greater attention to the user, more efficient support services, more opportunities for the user, and an increased support for human interactions. People are surrounded by intelligent and intuitive interfaces, integrated in every kind of object, and by an environment that is capable of recognizing and responding to the presence of different individuals in a fluid, unobtrusive and often invisible scenario [12]. The major characteristics of a smart environment from the point of view of a user should be: not intrusive, personalized, adaptive and predictive. The creation of a smart environment offers new possibilities of integration but at the same time opens new challenges regarding the usability of products for people with disabilities [13,14,15]. The problems of accessibility are not yet well defined, since future lines of development of the information society are still open with regard to various aspects: type of technologies that will be used to build the new smart environment; type and nature of the new applications and services that will emerge; contexts of use which will extend the Information Society; strategies put in place to extend the use to all potential users. However, there is a growing evidence that the accessibility and usability within a smart environment by users with various needs and requirements cannot be obtained with ad hoc solutions. On the contrary, intelligence, integration, accessibility and usability must be essential components and constituents of new environments and this is the purpose of HDOMO project. AAL and AmI systems are becoming simple and accessible, both for end-users and for installers, thus causing a growth in the market and gaining, also in Italy, a wider audience. Our country is the third European market for home automation systems after Great Britain and Germany, with a market share of 11% and approximately a 18.5 million euro turnover, of which 20% is targeted at AAL/elderly. This is a growing market and many funds have been allocated by the Italian State. In particular, the Italian State created AitAAL (Italian Association on Ambient Assisted Living), an association with the aim of providing innovative solutions to improve the quality of life of older people. Prices of home automation systems are decreasing, thus allowing a further extension of the market. The global drop in prices is due to several factors, including increased competition among players operating in this market, advancement of knowledge in some key technologies (such as ICT), and growing economies of scale allowed by the progressive enlargement of the market. This last aspect triggers a vicious circle between cost reduction and enlargement of the market that is already happening in some areas of AAL and AmI.

Another area related to the HDOMO project is linked to the Internet of Things (IoT) paradigm [16,17,18]: objects become smart, are localized, can acquire data, process and exchange them. Smart home and building applications are particularly important for the IoT scenario, as they represent the link between the individual (citizen, consumer) and the overlying layers of adoption of IoT paradigm (Smart City, Smart Grid). The most important technological leaps of the project are therefore: integration of functions/tools/services, interoperability, standardization, accuracy based on multiple SO alarms and, finally, accessibility.

Personal security is an important aspect of the project, because the system not only provides the traditional alarm system, but also offers a control on the activities of the users, as well as intrusion detection using the latest domotic technologies (face recognition, presence of movement, sound identification). This security aspect is both instantaneous and adaptive. Instantaneous because it is possible to instantly identify if one or more users have medical problems, thanks to sensors that detect physiological parameters (including the breathing and postures) and to audio sensors. The system immediately recognizes anomalous situations and, through its communication systems, interacts with qualified personnel to intervene and to offer assistance to the user in distress. Adaptive because an intelligent algorithm learns the customs of the users over a long period, so a change of these customs can have a high added value to the medical anamnesis of each user and her/his state of health, by allowing the identification and treatment of health problems. The system also provides a security environment as it can handle any home problems (electrical short circuit, hydraulic leaks, gas leaks, fires and so on), promptly alerting users or whoever takes care of them and, if possible, proactively acting to correct the problem. The required sensors will be: microphones, cameras, air quality sensors, piezoelectric floor, RFID, smart appliances and sensors for health monitoring. The system has a high degree of integration with basic home automation and is interfaced with the most common home automation standards and their recent innovations. In AAL applications, where sensors are often used to retrieve information about the assisted person, it is very important to consider privacy and ethics issues. This can be problematic when sensing technologies are involved, in particular, vision based approaches. In order to prevent the subject from being visually identified by any images or video of the scene, the vision system must be modified in respect to the privacy. The application of ethical rules and clarity in the use of data are fundamental principles to increase user acceptance. Jia Zhou et al. [19] demonstrate the relevance to include diverse user groups (age, diseases, disabilities) and their specific needs and wishes into the design and evaluation process of AAL technologies.

### 1.4. Innovation of HDOMO

**Figure 1:**
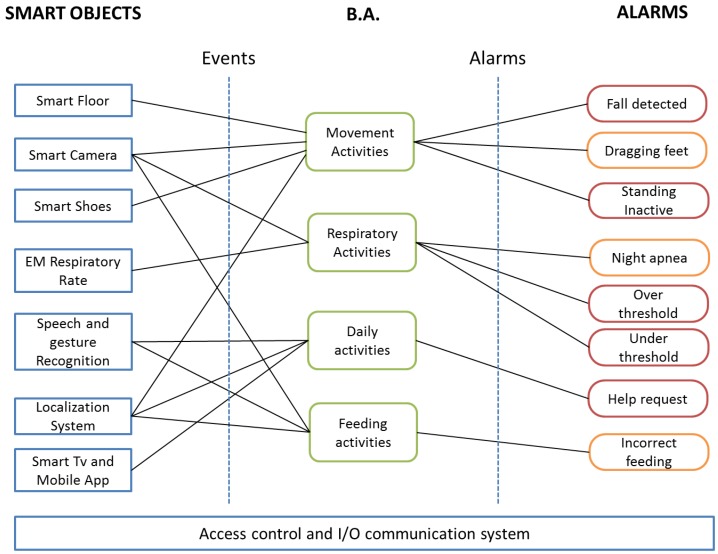
HDOMO logic architecture: 7 different SOs, based on multiple sensors, process data at low level to perform 4 different Behaviour Analyses (B.A.), which are processed at high level in the cloud infrastructure and are able to generate 8 different alarms on two levels: level 1 (critical alarm—red) and level 2 (non critical alarm—orange).

Figure 1 shows the general logic architecture of the HDOMO framework. The main contributions of the proposed project and paper are on the ability to mix different highly accurate SOs on a real scenario. Seven different SOs, based on multiple sensors, process data at low level to perform four different Behaviour Analyses (B.A.), which are processed at high level in the cloud infrastructure and are able to generate eight different alarms on two levels: level 1 (critical alarm—red in the figure) and level 2 (non critical alarm—orange in the figure). This architecture allows a high level of accuracy and reliability for a system with its main innovation in the intelligent data fusion and interoperability among different low cost SOs. The architecture is flexible and reusable in any AAL project where there is more than one device capable of monitoring the same behavior or activity. One of the big problems in AAL projects is the lack of accuracy in recognizing behavior because the monitored subject can perform it in many different ways. In addition, accuracy decreases because the device must not be invasive for the monitored subject. Through the use of cross data and sensor interoperability, it has been possible to increase the accuracy and reliability of the entire system.

## 2. Related Works

In this section, we briefly describe some projects related to HDOMO. This list is not exhaustive, since in recent years there have been a lot of projects providing innovative ideas for ambient intelligent applications. To have an overview of works and projects and to get an idea of the state of the art concerning smart environments see the works of Chan et al. [20] and Alam et al. [21]. They make a review of smart homes considering different aspects: comfort, healthcare, security [21], and arranging the projects according to country and continent [20]. The sphere of home automation and smart home suffers due to the wide variety of specifications and standards that do not allow communication among different devices. Considering also previously cited works [20,21], this problem is being tackled on several fronts: there are several research institutions and government agencies that have developed frameworks for the management of networks in intelligent environments. Below, we cite the main developed frameworks. Blasco et al. [22] proposed a Smart Kitchen implementation which provides AAL services for elderly and disabled people; it is based on a modular architecture based on a OSGi that makes it possible to build a complex system composed of small modules devices. The system concept and its implementation are innovative, merging many different technologies to build the smart environment: RFID technology, wireless sensor networks, distributed computing, artificial intelligence, etc.

The Energy Conservation and Homecare Network (ECHONET) (https://echonet.jp/english/) is a standard for controlling in home automation devices (air conditioners, lighting devices, and so on) and connects them through a gateway device. The specifications provide the use of APIs and standard protocols to promote the development of applications and define an open architecture. The main purpose of ECHONET is to apply ECHONET specifications to the home energy management system, defining an ECHONET object for a smart meter, and ECHONET properties for energy management on home appliances.

Most recently, the Digital Living Network Alliance (DLNA) [23,24], an international cooperation between computer industries and companies of mobile devices (over 250 companies), has developed a common standard for video sharing and other digital content created by home appliances, personal computers (PCs), and mobile devices. The specifications are mainly based on existing standards, such as standard Internet TCP/IP protocols to connect the devices, and Universal Plug and Play (UPnP) for resource sharing.

Ishikawa, in his recent work [25], highlights the issues of the standardization activities mentioned in this section, from the mobile phone and cloud server points of view. The author affirms that such technologies control and manage specific home appliances, and so there is a lack of technologies and standards for controlling and managing heterogeneous home appliances in a consolidated manner. To solve these issues, the author proposed overlaying networking protocols and metadata technologies as a solution for controlling and managing heterogeneous home appliances connected to home networks. For this purpose Peer-to-Peer Universal Computing Consortium (PUCC) was founded to deploy an architecture oriented towards de facto standardization. The European Union has invested heavily in ICT for Ageing Well projects in recent years. The programme is part of Horizon 2020, the new Framework Programme for Research and Innovation (it has a total budget of 80 billion euro from 2014 to 2020). Some of the main projects are OPPORTUNITY (http://cordis.europa.eu/project/rcn/89026_en.html), RUBICON (http://cordis.europa.eu/project/rcn/97731_en.html) and DOREMI (http://cordis.europa.eu/project/rcn/94439_en.html).

## 3. Description of the Architecture

**Figure 2:**
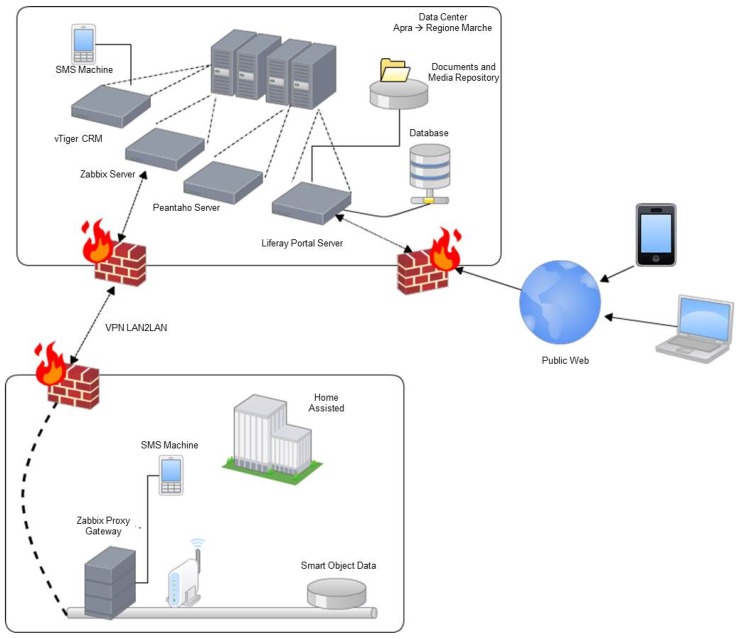
General data processing, maintenance and security architecture of HDOMO project.

**Figure 3:**
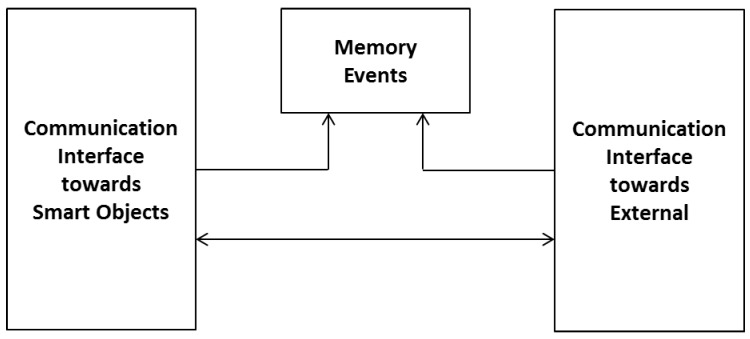
Block diagram of Communication system (ref. Figure 2, Zabbix Proxy Gateway).

The HDOMO project offers an agent and agent-less monitoring, scalable, distributed and extensible architecture able to connect smart objects that use different standards. This allows to add any home equipped with a smart object to the centralized platform. Figure 2 shows a simplified representation of HDOMO data processing, communication and security architecture. It consists of two main functional blocks: the first identifies HDOMO home and the second identifies the AAL as a Service (AALaaS) platform of HDOMO project. The platform is based on a virtualization infrastructure that uses VMware ESX Server, which offers high availability. The communication between the home and SaaS platform is protected thanks to a Virtual Private Network (VPN) connection, ensuring a secure transmission of data. The main nodes of the platform are:

- Monitoring server based on Zabbix (www.zabbix.com);
- Contact management server based on vTiger;
- Presentation server for aggregate data that provide remote management of homes (based on Liferay);

Liferay platform, CRM vTiger server and Zabbix server are all integrated with a Single Sign on system based on a Central Authentication Server (CAS). These nodes are flanked by Analytics services based on Pentaho Data Integration (the classification system based on Weka), and by other nodes, which provide supporting backup and data restore services, and send alerts via SMS. The architecture provides the development and the integration of different devices introduced as SOs, which must monitor and support users in their daily activities. The project provides also devices able to detect anomalous situations and eventually interact with the user or send a distress alert. Each SO has different functional feature s and so different ways and technologies to access the information collected during normal activities of daily life. The communication system has to create a link among different objects, by promptly and completely providing all the information detected by all objects. Information that must be managed in real time belongs to the alert signal category, while signals which need longer time to be processed belong to the interactions category. Typically, information concerning interactions between the user and the surrounding environment can be more complex and voluminous and for this reason it is necessary to use a mass memory to save a large amount of data, and it can be externally transmitted when necessary (Memory Events in Figure 3). Another important feature of the communication system is its modularity, necessary to manage systems different for the number and the categories of SOs. This aspect makes it possible to introduce even if they were not part of the original plan, and build systems tailored to the characteristics of the environment and the real needs of the user to be assisted. After having received information, the communication system makes it available to the outside using standard technologies compatible with most popular communication services.

**Figure 4:**
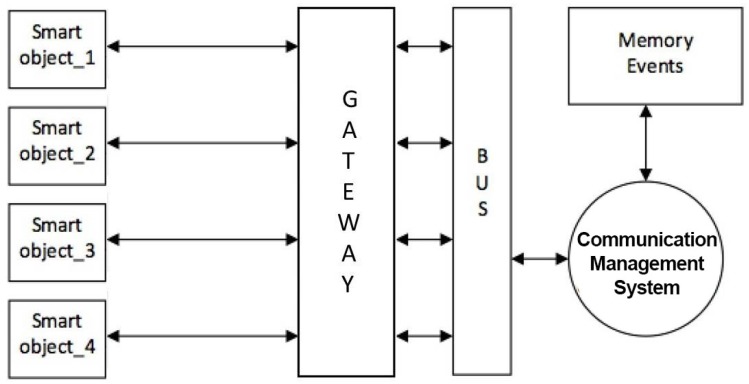
Block diagram of communication interfaces towards SOs (ref. Figure 2, Home assisted).

Figure 3 represents the communication system composed of three main blocks that together manage alarms, interactions and communications towards the outside. SOs responsible for the detection of dangerous events are considered by the communication system as alarm signals and therefore have the highest priority compared to all other types of reports. In the analysis of the management of alarm signals dangerous events have been identified as most probable and most important:

1. falling down;
2. irregular heartbeat;
3. fire;
4. flooding;
5. intrusion of strangers;
6. distress call;
7. excessive humidity in the environment;
8. malfunctioning communications systems;
9. therapy-mandated drug delivery;
10. anomalies in the habits of the assisted;

The first five kinds of events are considered active because smart objects enabled to recognize these events trigger automatic alarms when the event occurs and the sixth event provides an explicit request from the assisted and is therefore considered passive. The last four types of events are also active and are reported after a variable observation period, and therefore considered to be slow. These are the possible alarm systems: light and acoustic signals, phone call, sending of SMS messages, sending e-mail and audio-video connection. The light and acoustic signal involves a very limited zone but it can be useful when there is the operator responsible of the assistance nearby. The phone call is the most easily-implemented system and it can manage both the distress calls and the step of interaction between the patient and the operator in preparation of the assistance intervention. Sending SMS messages is easy to manage but does not always guarantee the immediacy of information delivery and does not offer the interaction with the patient during the preparation of the intervention. Sending SMS messages can also handle very complex information such as photos and video but this is not a problem for the operators. Audio-video connection is definitely the most complete but binds the operator and the assisted to the use of expensive technologies and communication channels that are not always available in all areas. Interaction management provides the exchange large amounts of more complex information concerning events such as posture of the assisted, position of the assisted inside his home during the daily life, as he interacts with the television and other home appliances, if he receives visits from other people, and so on. To manage a large amount of information a local system that can store all the events and when necessary to transfer all the information to an external processing center has been used. Considering the various technologies and existing systems which provide information transfer, the communication system must be arranged so that it can accommodate all the technologies provided by SOs as also chosen in a particolarly installation. Such a general system it is expected to identify a technology that can handle both large and small amounts of data and that has a high transfer speed. Observing Figure 4, the interfacing between the SOs and the management system of communications called gateway provides both the direct connection compatible to the standard used by the SO and the connection to the management system of the communications through a pre-defined bus standard.

**Figure 5:**
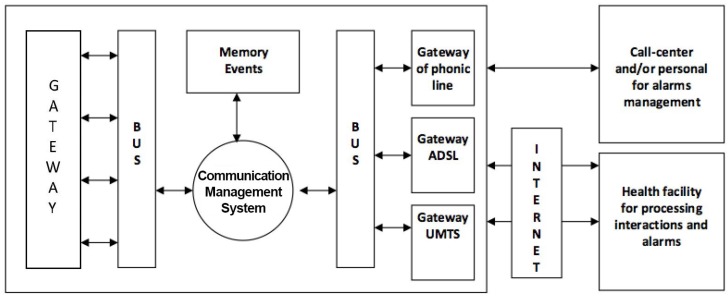
Block diagram of communication interfaces towards the outside (ref. Figure 2, Entire).

Depending on the type of information to be transferred to the outside, two main types of communications are defined: phonic and data. The communication channels available for the management of various types of communications are: fixed telephone network, mobile connection, and the Internet. Phonic communications are used for reporting and managing alarm events directly addressed to operators or friends or relatives of the assisted that can receive calls via landline and/or mobile phone. Data communications include both large amounts of information about the historical of everything that has happened in a day, video, audio, and so on. The telephone network and/or mobile phone are primarily used for phonic communications and towards service centres such as call centres that handle the emergency. For the management of communications between the gateway and the communication system, standard ethernet found in all computer networks both wired and WiFi has been used, for the following reasons: is very common, is a worldwide standard, provides very high data transfer rate up to Gigabit and beyond, provides both wired and wireless connection and the components necessary to realize the interfaces are easily available at very low cost. For connection to the outside, standard interfaces of telephonic line have been realized while for data communications have been used ADSL standard modem and for wireless connections UMTS modules as Figure 5 shows.

The gateways that connect SOs and interfaces towards the outside have been directly managed by the communication platform. For the manage of phonic communications towards operators, friends and relatives, a reduced communication system have been developed that can help the main platform.

### 3.1. Distributed Monitoring: Zabbix

Since the project includes a 24-h monitoring of the home and so of the SOs, to verify the correct functioning of objects, applications, and in general services, Zabbix has been chosen as monitoring software. Zabbix is a centralized monitoring software of server systems. It is a classical agent-server based architecture. Each system to be monitored can be equipped with an agent that allows a very precise and constant data collection since it is continuously connected with the main monitoring server Zabbix. Several are the advantages of Zabbix, among these we listed: agent and agent-less monitoring, scalable and distributed architecture, easily extensible with plug-ins or agents, storage for monitoring data based on database, opportunity to generate real-time graphics with instant alert, and others (in Figure 4 SOs are equipped with Zabbix agents that send data to the proxy Zabbix server installed in the gateway). The flexibility of Zabbix is an important element that distinguishes it from other existing open source solutions as it allows virtually monitor and intervene on any element of the infrastructure through a various number of standard protocols (Zabbix Agent, Agent-less, SNMP v1/v2/v3 & trap poll, Log parsing, ODBC, Java, SSH, Telnet). It also allows the acquisition of data through custom scripts definable by the user. Zabbix provides a very scalable monitoring system that starts from a traditional centralized solution to complex distributed architectures on multiple control nodes and proxy data acquisition, this solution is useful for HDOMO project, where complex systems interface. Some SmartObjects not allow you to install the Zabbix agent inside them and also the Zabbix Proxy prevents a two-way communication with the server (you can send data to the Zabbix server, but you cannot receive commands to be applied on SMs). To maintain a high degree of interoperability gateway, it was added a module that contains drivers for SMs, and a module that provides Rest/Soap services to enable even these SMs to establish communication and exchange information with the Zabbix server. The module is called Planet Automation. An SM that requires registration through rest/soap services must provide the following information:

- Type of MS (recognition identifier);
- Protocol version with which began bordering;
- Any user identifier associated
- Lease time to update your subscription

To assess whether the web management system is available, a check will be made when the system boots to the predetermined endpoint.

### 3.2. Data Management: CRM vTiger

CRM server handles personal data of homes and users. A required information to be loaded into the CRM is the telephone number of the SIM installed in the BOX of the home; additional information can also be managed, such as: personal mobile phone number and other information. For each user information about the “Contacts” are managed, people that are referents: family, doctor, doctor on call number, 118 and others. For each referent must be inserted a telephone number, since CRM is connected to a VOIP switchboard. The reception of a call or SMS implies the automatic opening of a Ticket, which consists of the following information:

- First and last name of the caller (automatically proposed);
- The contact associated with the user;
- The list of available contacts, with highlighted phone numbers, associated to the user;
- The reason for the call;
- The identification data of the operator who is completing the Ticket;
- The status of the Ticket;
- The ability to manage the progress of the state, a number of states of the ticket will be the default: Open, Check, Waiting response, Closed, and so on.
- For each change of state is required to enter a description and, automatically, the operator ‘logged in’.
- Managing the priorities of the ticket
- Possibility of passage of managing tickets from one operator to the next
- Wherever it is visible a contact telephone number, the system allows a management as ‘Click to Call’, the ability to quickly call a number by clicking on the number displayed on the screen.
- There is a link to the documentation of the operating protocol for the operator.

The CRM has thus been customized to handle the typical entities of a project AAL.

- The registry of the house.
- The registry of users;
- The registry of SO (to exploit the capabilities of asset management of CRM).

### 3.3. Data and Alarm Consulting and Process Management: Liferay

The project uses the Open Source Liferay platform for system management, provisioning, alarm management and presentation of information. Liferay is a platform focused on the aggregation of resources and generally to applications and services. The platform can aggregate communications deriving from SOs, process them according to the precise specifications by giving automatic alert facilitating the realization of services and intervention. Liferay supports the mechanism of workflow and “out of the box” by providing the Kaleo Workflow engine. It is available through a portlet. It is used during the testing phase, but successively it can be replaced using the most functional and flexible Activiti process engine.

## 4. Development of Alarm and Interaction Management System

Two partner of the project (Eurosystems (www.eurosystems.it) and Rico (www.ricoitaly.com) developed a communication interface both phonic and data to the outside that uses GSM and GPRS technology and a cloud service for programming and monitoring interface and transferred information. The choice is a hybrid solution on mobile network since comprises all required requirements (phonic and data communication). The project consists of two types of interfaces:

1. GSM-GPRS communication interface;
2. Cloud service for remote management of interface;

The interface developed provides the following main characteristics:

- GSM-GPRS quad band module;
- phone line simulator;
- USB 2.0 port for data communication;
- power input;
- Managing backup battery.

**Figure 6:**
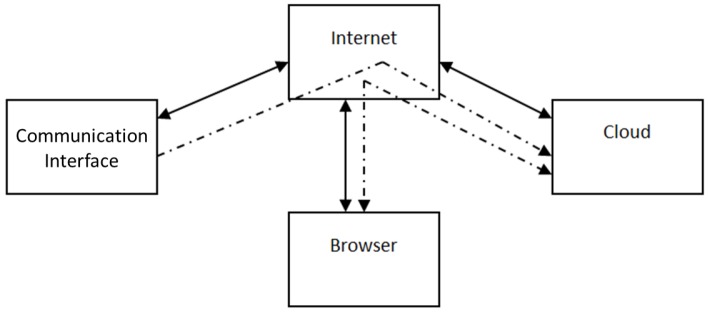
Block diagram of the communication scheme between data derived from SO and the external world.

To simplify the management of the main functions of the interface and to monitor information detected by the communication platform has been developed a Cloud service accessible through any browser. Figure 6 shows the diagram block of the communication scheme between data derived from SO and the external world.

### 4.1. Communication Interface

The interface uses a GSM-GPRS quad-band module able to manage audio and data communication. For the connection towards communication devices such as telephone, dialer, and so on, an interface of standard telephonic line has been developed, while for data communication has been provided USB 2.0 port. The interface is alimented with 12 Vdc and also provides a battery input that allows its functioning also without power grid. Through the USB connector it is possible to connect the communication system that gathers all the information from SOs. The communication system manages the interface for different types of functioning.

### 4.2. Cloud Service

The cloud service has been developed in order to manage all the functions of the communication interface and to control the transferred information. Compared to traditional management systems of technological devices, Cloud service has several advantages that allow to simplify the functioning for its management. In fact, it is not necessary to install a specific software; it is possible to use a smartphone, tablet, pc or other devices that have access to Internet and to use a browser; wireless connection occurs in local or remote. The service has been developed using Apache server, Mysql database and the software has been developed using PHP, HTML and Javascript languages. From the home of the browser is sufficient to type in the Internet address of the portal of Cloud management and then a form is opened where the user has to insert username and password. Once inside the private area of the communication interface, it is possible to use all the intended functions:

- Description of the installation: name, location, and so on;
- Functions: telephone numbers where to send text messages, schedule for synchronization, sending messages to the interface;
- Monitoring the status of the system: the state of the SO, the last transferred data, and so on.
- Logging of the events activated on a Web server;
- The interaction management provides for the exchange of information much more complex and in large quantities concerning various type of events such as the posture of the assisted, the position of the assisted.

## 5. Interoperability among Heterogeneous Devices: Architecture and Software of Hdomo Gateway

HDOMO 2.0 project fits into the areas of: Home Automation (HA), AAL and AmI in order to create an integrated environment, with several smart devices, able to assist weak users within their own living environment. So, it is important to integrate and connect multiple hardware devices: domotic devices and environmental sensors that are in the infrastructure. The actual scenario presents both the absence of standardization protocols and means of communication for interoperability of home appliances, and high costs of hardware to obtain these functions incompatible with cost requirements typical cost of the market. However, during the years, the development of electronics allows to have a great number of sensors and hardware devices at a very low cost in order to realize several AAL applications. If on one hand this is a positive boost to the development of custom applications that make use of embedded sensors, on the other it is necessary to take into account that the producers of different devices, provides together with the hardware, also software and interfaces to be used to communicate and to extract useful information from them. In most cases, the latter are digital electronic devices, with a structure that makes it impossible a direct connection of these custom sensors with the domotic component of the system. In the context of AmI, the processing unit has to integrate all the information extracted in intelligent way from hardware devices and, to this end, interoperability becomes a key requirement. To make the correlation among such information, a high processing capacity is required higher than the necessary for the physical management of hardware devices (sensors, actuators and HA devices). In HDOMO project, the gateway will connect the following communication bus:

- Towards digital devices: Ethernet, SPI, I2C, UART, RS-232/485;
- Towards hardware devices;
- Predisposition towards HA standard;

These premises have led the development of HDOMO gateway, which will be described in the following sections, aiming at the complete integration of different elements in the project. The communication between the main unit (i.MX6) and the secondary unit (K60) occurs through UART, with 19,200 bps baud rate.

### 5.1. Hardware Design of Gateway

Hardware design of Gateway includes following steps:

- Functional Design: following the analysis of the requirements have been identified circuital solutions and the individual hardware components can be used to realize the gateway device;
- Electrical Diagram: it is made the electrical diagram of all custom electrical parts to be included within the gateway;
- Routing, and prototype implementation of the PCB board;

The gateway can be connected with a smart modular digital camera additional feature. The high computing capacity of the gateway also provides the possibility of integrating a vision system developed by other project partners. The device consists of two processing units, next described.

#### 5.1.1. Main Unit: iMX-6 Platform

**Figure 7:**
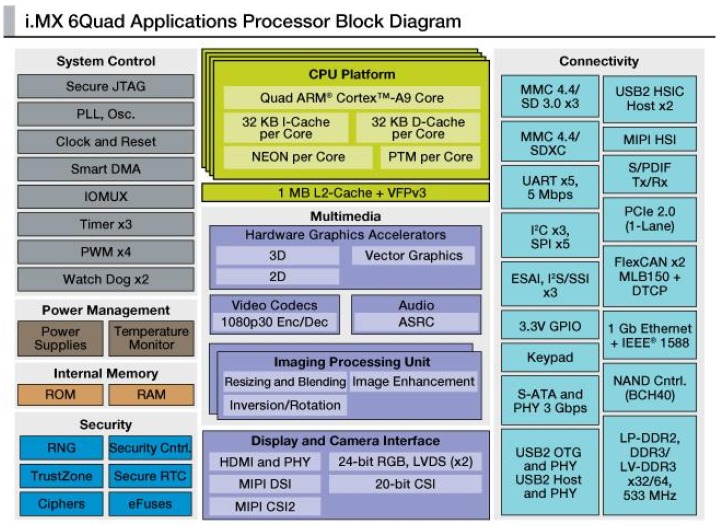
Architecture of the processor used in the main unit of gateway.

The primary unit is used to manage general-purpose functionality and for all that concerns the management of high-level and computationally onerous tasks. To do this, as hardware platform has been used a commercial board based on IMX-6 processor of Freescale. Figure 7 is a general overview of the architecture of the processor chosen.

The main unit presents high scalability of communication: among its standard interfaces are 1 Ethernet Gigabit port, 2 USB 2.0 port and a Wi-Fi interface. Concerning serial connections, this component has 3 serial port, each usable as RS232/RS485. Taking into account this adaptability, these choices are done:

- number 1 RS232, predisposition to interconnect one bridge device (commercial and external to the gateway) with commercial domotic bus;
- number 1 RS485, predisposition to effect multi-point interconnection, by increasing the number of possible connectable devices;

The main functions by this processing unit inside the gateway are:

- Integration of information coming from the secondary unit and from devices its connected;
- Execution of calculations algorithms necessary to process informative flows;
- Manage of interactions from and towards external environment with the internal network of HDOMO.

#### 5.1.2. Secondary Real-Time Unit

**Figure 8:**
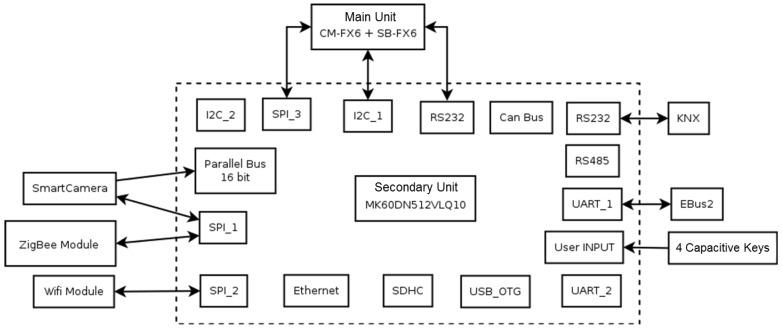
Block diagram of secondary unit interfaces of the HDOMO gateway.

The block diagram of the interfaces that are on the secondary is shown in Figure 8. It is aimed at lower-level tasks and management of device-set characterized by particularly stringent time constraints.

The micro-controller has been selected for its particular features, among which:

- Freescale provides for micro-controllers of Kinetis family a real-time operative system, MQX RTOS;
- K60 family has hardware and software support necessary to create web server and applications with HTTP protocol;

In particular, the secondary unit provides:

- Hardware predisposition for interfacing with domotic part of the house using commercial domotic bus;
- A method to manage low-level hardware devices, characterizing from simple communication protocol and/or with stringent temporal characteristics, as previously said;
- Hardware predisposition for interfacing smart sensors, as for example:
  - Breath sensor: that allows to detect the frequency breath of the assisted, sending detected data through RS232 connection;
  - CO sensor: that it is responsible for detecting any presence of carbon monoxide. It is connected to K60 board using analogic input and its value is converted through ADC (the sensor is CO $click^{TM}$ able to detect levels of carbon monoxide between 20 and 2000 ppm).
  - Temperature sensor: it is responsible to measure the temperature of the room, and it is connected with K60 board using analogic input and its value is converted through ADC.

**Figure 9:**
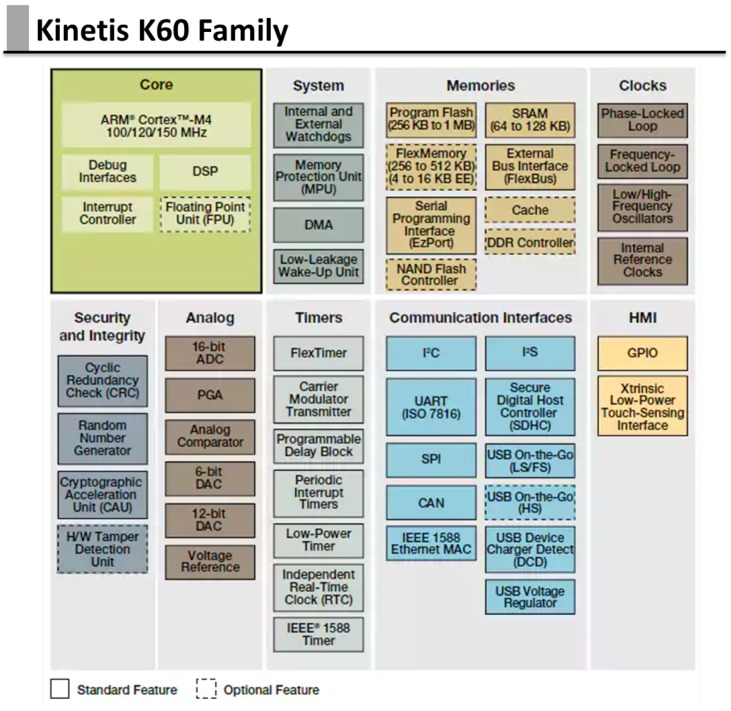
Architecture of K60 micro-controller used in the secondary unit of gateway.

Figure 9 is a general overview of the architecture of k60 micro-controller chosen.

### 5.2. Software Design of Gateway

From the project point of view, the software of the gateway consisted of the following macro components:

- Application level software, for the main unit: contains the implementation of the high logic level. It has been executed as application inside the operating system of the main unit and so its implementation depends on it;
- Operating System of the main unit: Linux system has been installed on available to us board and some driver have been installed: Ethernet, serial interfaces, I2C bus, SPI bus, and so on;
- Firmware of the second unit.

These software components will physically implement the logical model of the gateway system. Inside these three components coexist subsystems of communication, processing and control.

#### 5.2.1. General Architecture of the Software

The software has a modular architecture subdivided on different layers, by organizing different components according the state of the art of the most important international standard in the field of software engineering. The main advantage of the stratification is to allow the abstraction of different levels of program, departing from the hardware of the micro-controller until to arrive to different services of the Operating System and to communication interfaces. This kind of approach allows to write high-level application modules that are as possible as reusable and general. Modularity and stratification determine the effective scalability of the application: the project is configurable basing on actual requirements that can be dynamically changed, allowing the running of the software transversely on several design options. The software has also the advantages to be reusable and portable. The first advantage allows to also reuse software modules written by other developers. The API standardization on different levels of abstraction and the structuring of the software will allow to write modules in a standard way, with homogeneous interfaces towards lower and higher levels. This will lead to a significant improvement also in terms of quality and efficiency. The second advantage allows to freely change HW and SW platform without compromising the functionality of the application. The API in input and output of the OS and the way to interact with it will be exclusively handled by a dedicated SW Layer, which can be changed without affecting application modules.

- Control system: its functionalities are made by module of events, alarms and report management and by modules of logging events and manage of non volatile memory. Several actions to be taken are then transmitted to the below layer to be operationally performed, as the generation of an alarm, the transmission of a message on the network or the saving of occurred events.
- Communication system: the functionality of encoding/decoding datagram module and all single communication modules. This software components allow the communication among different devices and their integration on the gateway.
- Audio/video processing system: its functionalities are inside the acquisition modules from smart-camera and stream video processing. Software modules of video processing are treated in a separate way from all the others.

**Figure 10:**
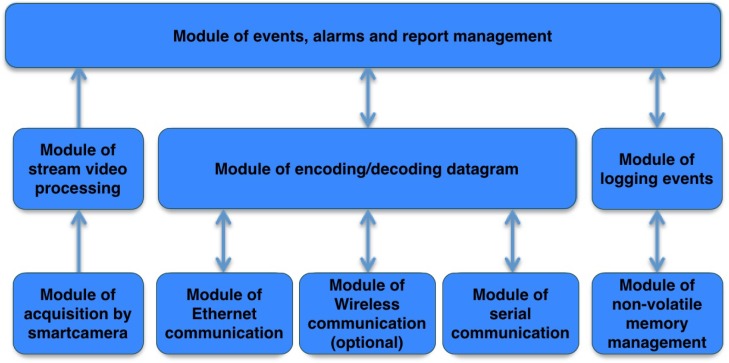
General architecture of the software.

Figure 10 shows different modules that are described following in detail:

- Module of Ethernet communication: it sends and receives Ethernet messages from HDOMO actors that uses this bus to communicate on HDOMO network;
- Module of serial communication: it sends and receives data through serial connections available on the gateway platform (RS232, RS485, UART, SPI, I2C) from HDOMO actors that use this bus to communicate on HDOMO network;
- Module of datagram coding/encoding: it is responsible to delineate the semantic of messages coming from various communication channels. It receives HDOMO commands from the outside and forwards them to the below correct module, that will properly transmit on the network. Information that come from several devices are extracted by this module and then forwarded to module of events, alarms and report management.
- Module of events, alarms and report management: it receives and forwards events and alarms. It dialogues with HDOMO portal and connects the external world with domestic network.
- Module of logging events: it manages the saving of detected and generated events. The logging module receives as input data to be saved from events, alarms and report management module and manages non-volatile memory through this module.
- Module of non-volatile memory management: it operationally manages non-volatile memory of the gate.

Each software module is divided into sub-modules, developed for the mail and the secondary unit of gateway. The task of the secondary unit will be physical management of components its connected.

## 6. Smart Objects

The Hdomo project involves 16 small and medium-sized enterprises and 2 research institutes. Each of them has offered their own knowledge and expertise at the service of the project. A series of intelligent devices have been defined to be developed in every field of competence of the actors involved in the project. Each smart object can work independently but is designed to be easily connected to the HDOMO architecture. In the supervision system are developed and integrated the following SOs:

A Localization system;
B Smart floor with energy harvesting and localization functions;
C RGB and RGB-D smart cameras for the analysis of the presence, of the postures and of the interaction between user and environment;
D Smart shoes with localization functions and analysis of motor activities;
E Smart tv and app for devices management and the information;
F Audio analysis system for speech recognition and novelty detection;
G System of gesture analysis;
H Non-invasive analyser of respiratory rate;
I Videocitofonia system for easy access at home in an emergency situation.

The following subsections are dedicated to describing in detail each integrated object as part of HDomo project.

### 6.1. Localization System

**Figure 11:**
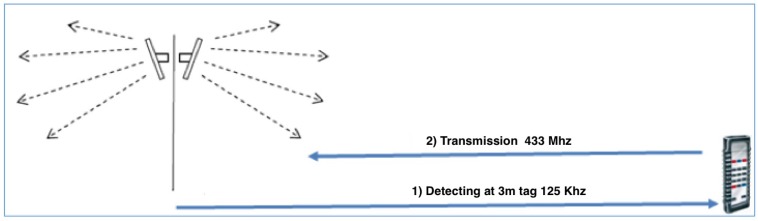
Communication between the transponder and the antennas.

Having identified the antenna and frequencies system most appropriate for HDOMO project (Figure 11), the next phase was the integration of information coming from the localization system with the platform of building automation. The technology chosen to localize a person within the home, the small size of the semi active transponder (about 3.0 × 2.5 × 0.7 cm) have facilitated the work of engineering and have allowed the inclusion inside the command and control device (remote control) without great difficulty. The choice to insert the tag in the remote control derives by the fact that the device is always brought with him from elderly or disabled person (if placed on the wheelchair or placed in the neck) so Planet Automation platform is able to know at any instant the position of the user and is able to handle the information obtained by the localization system. By means of the standard Bluetooth, the remote control communicates with the Planet Automation (the remote-control signals to the central system, via wireless transmission, its location and then that of the user).

The system of presence detection has to be able to know at any time the position of the person with the remote control and can instantly configure Smart Objects installed in the same environment. In this way, HDOMO system could automatically turns on or off a light when the user moves from one room to another, enable the electronic locks positioned for example in the kitchen, which otherwise might be blocked in the presence of children in the home. Precisely because of the possible benefits that may have to know the user’s location within the house it was also important to identify the moving direction. Therefore, to identify the direction and detect whether the transponder has crossed a certain area in input or in output, an installation with two antennas has been realized, connected to a control unit that based on the sequences of detection is able to send to the host, the exact information on the moving direction.

**Figure 12:**
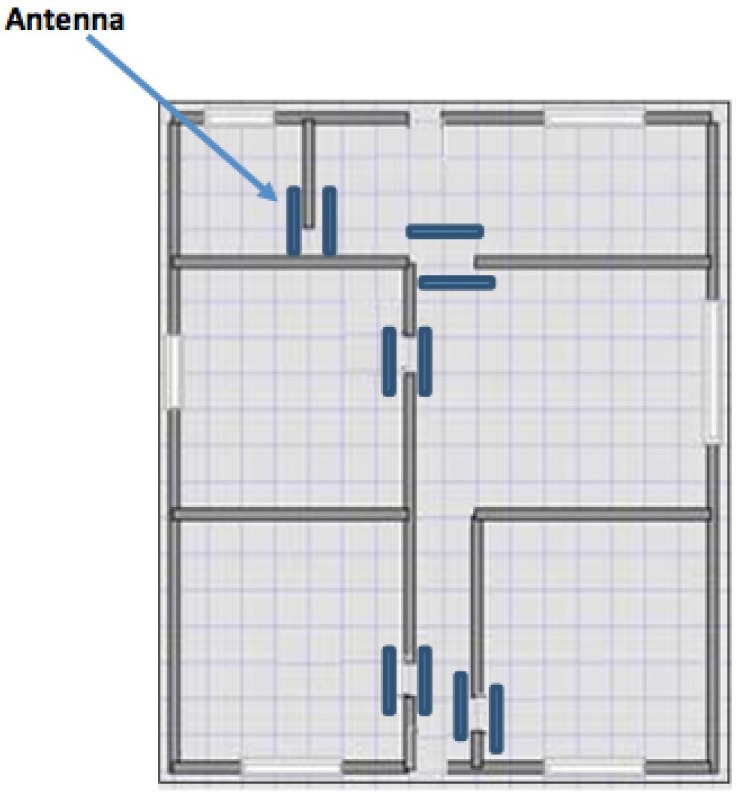
Position of antennas in the house.

The antennas are positioned in correspondence to the openings of the dwelling (as Figure 12 shows) for detecting the passage of user with RFID tags. The localization system tests took place in two phases: basic test of transponder reading with a field of action of the antenna set on two meters and advanced tests with verification of the transit direction recognition of transponder reading. The first test was necessary to understand the effective functioning of the antenna-transponder system. These tests have demonstrated that in most cases the transponder was read and it is possible to localize it. The reading of the transponder is not influenced by the mass of the human body and the field of action of antennas easily through walls and furnishings. The only reading difficulties are determined by the presence of metals that can shield the transponder. However, even in cases of failure to read, through appropriate software configurations, the system is able to restore the correct operation of the remote control at the time when the same is detected, even after a period of missing readings. Since the beginning of the research part of the study was oriented to verify distribution schemes of channels realized within the physical structure of the house, identifying as optimal solutions the following positions: counter frames of doors, counter frames of windows, horizontal perimeter connection between vertical wall and ceiling, vertical connection between ceiling perimeter distribution and below counter frames of fixtures. It was also completed the verification phase with local producers and applicators to evaluate cost, feasibility of use both in the case of new or existing building. The optimization and cost reduction insertion of home automation systems, can be done by identifying and “preparing" distribution schemes can accommodate, in time, new equipment and/or to modify those originally installed. The most effective proposal able to guarantee in the most rational and economic way an easy integration between the building structure, notoriously rigid, and the variability/flexibility of all the sensory systems both that to establish a type of distribution protocol of metallic structures already widely used, and in the new construction and renovation of the existing building.

### 6.2. Smart Floor with Energy Harvesting and Localization Functions

The realization of a floor that allows the localization and analysis of the movement of users, interests many different applications from those of the present project, the approaches for this are many and varied. Many systems have been developed to monitor the position and movement of people for various applications: motion capture (also for artistic and cinematographic purposes), security, interaction in virtual reality environments, and so on [26]. The floor must be considered as a multifunction device, whose functions are manifold: mechanical support, adequate response to the needs of heating and damping of mechanical noise, satisfaction of aesthetic requirements of the environment, all obviously with acceptable costs. The floor of a room can be transformed into a floor-sensor that detects and controls the behaviour of people and allows a collection of new support functions [27]. The detection of movement in a room can be achieved using infrared motion sensors, with ultrasonic devices, with camera system and image processing [28]. However, these devices have technical problems due to different light conditions, blind spots caused by room furnishings and, above all, require a significant computational work. Also, a very important aspect to be reckoned is the absence of privacy. Systems based on vibration sensors may have problems with falls low impact and suffer anyway of shadow effects [29,30]. The reduction of the waiting time after a fall is an objective priority. Furthermore, prevails the need for an immediate local processing of the signal with only transmission of the result. The falls are one of the biggest health risks among the elderly. In a study by Fleming and Brayne, 80% of people unable to get up after a fall has not used the system of emergency call. 30% of these have remained on the floor for one hour or more [31]. We should minimize the residence time on the floor after a fall. During the walking, a power of about 67 W for the movement of the lower limbs is used. A person of 60 kg has to apply a force of at least 588 N through the foot during walking. If this force is accompanied by 10 mm of deflection of the floor or shoe, then the available energy is 5.88 J and taking two steps per second, an available power of 5.88 W per foot. Obviously, if a greater amount of energy is extracted means not a passive energy but use part of the work overload by the subject monitored: effect “walking on sand” [32].

**Figure 13:**
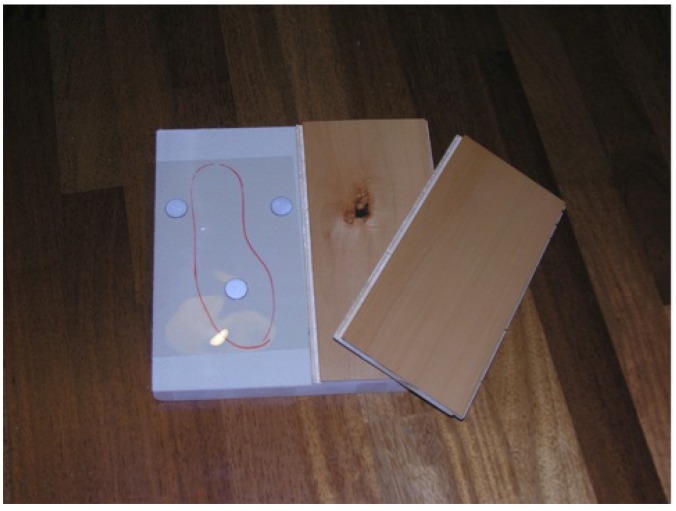
Capacitive sensors on polymeric support installed between solid wood and wooden part of a floating floor.

The smart floor pressure sensing systems have a number of characteristics that make it an obvious choice for user localization: users always walk over, it can sense information not only about users but also about objects. Thanks to the smart floor, the user does not need remember anything, it walks over the floor tile and the system utilizes biometric data pressure of the user for localization, tracking and fall detection. It is also possible to identify dangerous situations thanks to information on the pressure distribution on the surface of the floor and in the time. The smart floor also works fine when the room is noisy and dark, and it does not care if a view of the user is occluded. In addition, due to its very nature the floor gives accurate position information. In general, the algorithms for identification and tracking are simple and not computationally intensive [33]. In this project, we want to propose an innovative smart floor based on an energy harvesting system enabling the localization and analysis of the movement of the users in a specific space, also falling situations are monitored. This solution uses an innovative energy harvesting smart floor based on capacitive sensors situated on a polymeric support inserted between solid wood and the wooden part of a floating parquet as Figure 13 shows. The system is able to detect a falling using simple multiple sensor activations, by enabling to activate two different information sources: the indoor localization and the falling detection.

Inside the HDOMO project the system has provided data to the classification layer. Localization and falls data together with other data coming from HDOMO smart sensors are classified for HBA purposes by an activity model composed of Bayesian networks and support vector machines through a pre-processing phase and model training. The pre-processing module filters out noise, segments the data every half second, and extracts statistical features (minimum, maximum, average, median, standard deviation) from the smart floor data.

The system does not need to power the control unit save the task of collecting data coming from different sensors and processing all the information. The main critical aspect is represented by the capacitive measurement that is very sensitive. The cable is usually insulated with Teflon to address this problem. The simulation test of the floor functional prototype must be conducted with a load equal to about body weight (sinusoidal load weight equal to +30%) and with a frequency from 30 to 120 (maximum) distance to minute (0.5–2 Hz). The simulation reproduced the following different phases: Heel Strike-contact phase, Midfoot strike, stance phase, forefoot strike, or flaking, the initial one can last up to 60% of the entire cycle, this can be achieved by varying the height of the springs of the recliner plan.

**Figure 14:**
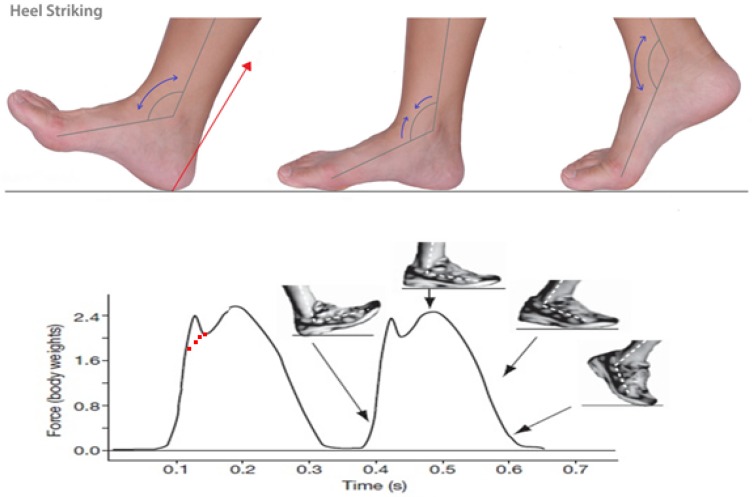
On the top, the movement of the foot. On the bottom, the forces exerted by different states of support.

Figure 14 shows different phases of the step. The top of the figure represents the foot showing the curvature angle. The bottom of the figure highlights the correlations between the forces exerted by the body weight and the instance of the step.

The contact angle between the plane of the sole and the ground is about 20${}^{\circ }$ and the contact force has an initial peak increasing from a normal deambulation (stance phase 60% of cycle time) to the running (stance phase 40% of cycle time). A medium person generally runs 120 steps per minute, the period per cycle is 1 s, while speed of 20 km/h the cycle time drops to 0.6 s, the contact phase changes from 0.62 to 0.2 s. The contact force increases until reaching a multiple of body weight increasing speed of the race. So the simulation test must be conducted with a load equal to approximately body weight (sinusoidal load equal to +30% body weight) and with a frequency from 30 to 120 (maximum) steps per minute (0.5–2 Hz).

It was decided to operate with sinusoidal load, with frequency 1 Hz avoiding the original peak since the shoes are suitable for normal walking and not for running. The shoe was mounted on a form with a joint that allows a rotation between the front and rear of the foot further details can be found in [34]). The contact angle between the abutment plane of the sole and the floor was 20${}^{\circ }$; by proceeding with the application of the load, the contact plane (ground) is lowered rotating around a pivot axis coinciding with intersection between the ground plane and the sole. This last is held in position by two springs in parallel by 60 kgf/cm and by further two springs (20 kgf/cm) intervened after the first centimeter of race to ensure the rigidity of the land. In this way, after heel strikes phase, due to the effect of rotation of the articulation of the foot and the lowering of the plane of contact, the entire sole supports (midfoot strike) and, for the load mode, the separation phase in which the forefoot allows to ultimate contact. The cycle was conducted with a load between 60 and 80 kgf. In order to check the effectiveness of the solution a number of simulations of use were performed. The guidelines adopted for these simulations are: to check that the possibility of not intercepting a sensor in two steps was negligible or very limited and to verify that, moving from any point, the possibility of intercepting a sensor is characterized by an isotropic distribution. In a daily life situation, when the assisted user falls down there is a multiple activation of the sensors and then the system autonomously sends an alert if the multiple sensors are not disabled this corresponding to a situation in which the person remains on the ground. The alarm is not sent when the person gets up again, and so the sensors are activated and then deactivated.

### 6.3. RGB and RGB-D Smart Cameras

**Figure 15:**
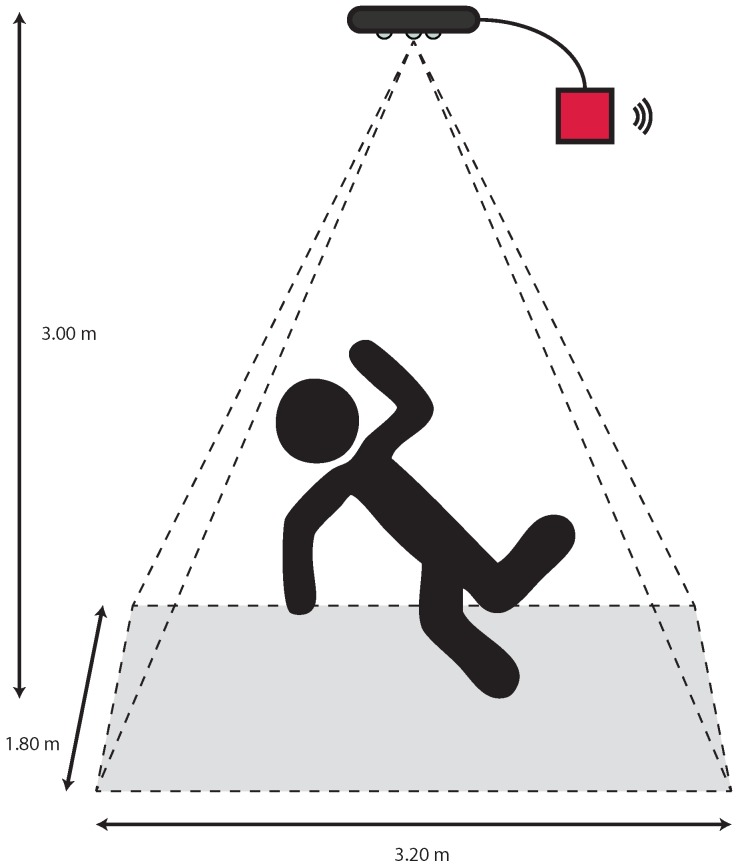
Representation of the RGB-D camera position.

Currently the most frequently used techniques for fall detection are focused on wearable sensors, such as gyroscopes and accelerometers [35]. The problem is that, these devices often generate false alarms, because some activities of daily living (ADLs) are manifested with fast moving down, that can be classified as a fall from a detector based only on inertial sensor [36]. In literature, a lot of research investigates fall detection methods using different sensors, see for example the review of Mubashir et al. [37]. There are methods based on vision systems, such as CCD cameras [38], specialized omni-directional cameras [39], multiple cameras [40] and stereopair cameras [41]. These methods present some problems about the respect of privacy and intimacy and they do not work in poor light or night-light conditions, that are the most which are among the most common situations in which falls happen. Hdomo proposes an automated RGB-D video analysis system that recognizes dweller activities that are crucial for assistance purposes, focusing the attention on the detection of falls. This work is part of a wider research work developed by one of the research institutes involved in the project. The algorithm has been adapted for the purposes required by the Hdomo project. For mapping the users to the floor plan and for identifying important events such as falls or sit in a chair we used low-level segmentation and tracking methods. By these observations, the system extracts a lot of statistical data that, after a processing phase, provide a knowledge base customized according to the needs of the users and the design of the home. The movements of the users are on line recorded using a database, so that their physical activity can be supervised anytime. Furthermore, the RGB-D camera is able to extract the depth images even in dark room, respecting privacy and intimacy with also re-id capabilities [42,43,44,45]. Observing Figure 15, the physical architecture of the system consists of a RGB-D sensor installed in a top view configuration. An embedded system manages the sensor acquisition processes the depth stream extracting measures of the people on the camera view. The sensor is installed 3÷4 m above the floor, by covering an area of 8.25÷14.70 m${}^{2}$. The sensor positioning, above the area to be analyzed, is more suitable compared with the front view configuration used for gesture recognition, in particular, the top view configuration allows to reduce the occlusions problems. In order to satisfy functional and non-functional requirements, such as the budget, we choose the CubieBoard21 embedded board. The board size is sufficiently small and energy efficient, and the computational power, suited to manage the computer vision algorithms. The Asus Xtion PRO LIVE2 has been chosen as depth sensor since it is cheaper compared to other depth sensors; it is smaller than the Microsoft Kinect3; it is powered directly by the USB port, without the necessity of an external power supply.

**Figure 16:**
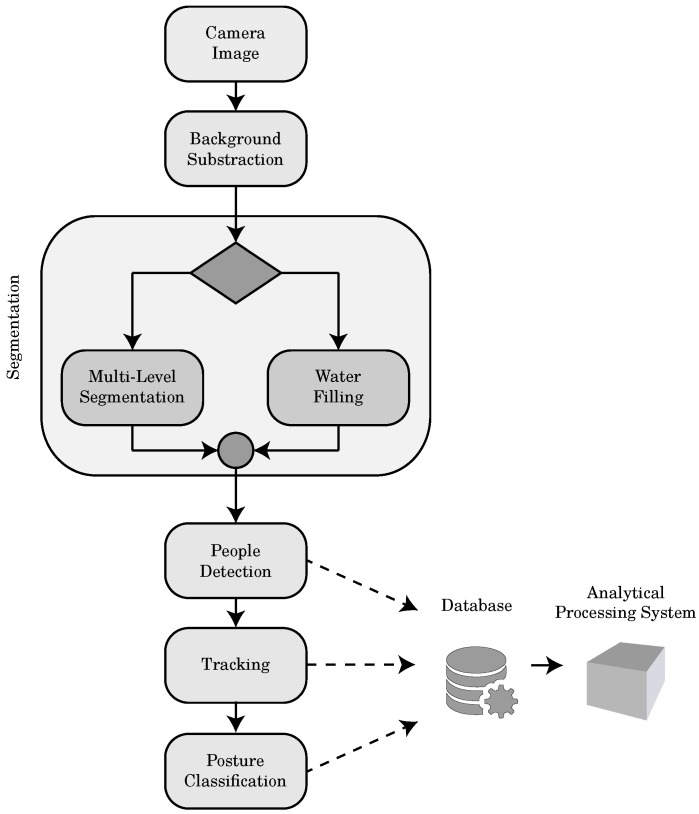
Block diagram of the implemented algorithm.

Figure 16 represents the main steps of the implemented algorithm that has, as input, a RGB-D image acquired by the camera. The sensor capture depth images, with a resolution of 320×240, at a rate up to 30 frames per second. The depth value is stored using 16 bit matrix allowing a spatial resolution in the order of millimetres. the acquired image is presented to the Gaussian Mixture Model background subtraction algorithm [46] that returns a foreground image that contains the people and, eventually, moving objects. The segmentation algorithm finds the heads of the people by filtering the image and exploiting the local minima of the depth image. This process is crucial for the correctness and the processing time. For this reason our system can be configured between two alternative algorithms: the multi-level segmentation algorithm [47] and the water filling algorithm [48].

Successively, people detection algorithm determines if a set of pixel represents a person or an object by using the features extracted by the segmentation procedure. The tracking algorithm recognizes if a person has been already identified by the system using the set of features: spatial positioning of the person and recordings of people recognized in a set of previous frames. If the person is identified, the system associates to the person the previously generated identifying label, otherwise it generates a new label and marks the person. In the room the heads of the people are tracked during their permanence in the scene. Measuring the height of the same person over the time, the posture classifier determines if a person stands up, is sitting or is falling. The last scenario is the most important for the elderly care and the robustness of this measurement, providing also an output signal that emits an alarm. A database registers the information obtained by the last processing. An Analytical Processing System, i.e., a separate process that accesses the data published on the database and extracts statistics and knowledge about the users. In order to verify the system capabilities in term of frame rate and accuracy, we have tested the system in a simulated environment, considering a bathroom, a kitchen and a hallway. The image acquisition and processing allows a processing rate of about 25 frames per second, meeting the system requirements. The accuracy test consists of 30 recordings of about a minute. For each recording, a number of people moves across the room with 4 chairs. The actors walk randomly and, for a total of 35 times, sit down or simulate a fall. The system is also to displays in real time the heat map of the movements of the people inside the home, that is a graphical representation of data where the values contained in the depth matrix are represented as colors. The proposed system is a valid instrument for the real time visual monitoring and for the registration of the people movements. Moreover, with our system it is possible to establish, with high confidence, the percentage spent in a certain region and to launch an alarm signal in case of a prolonged stay in fall position. The novelty of our work is its ability not only to recognize people but also to follow them in their activities, focusing on the analysis of their posture. Another advantage that makes it suitable to AAL purposes, is the ability to obtain results from the depth map of the images preserving the privacy of the person. The RGB-D camera was used also for the analysis of frail user activity and their interactions with the surrounding environment e with the objects inside it. As we said in the previous section, in order to detect by a RGB-D camera, user interactions with the environment a multi-level segmentation algorithm is used that allows people identification from a vertical depth image, even if people are freely moving in the room. For further details of the algorithm see [49]. The interactions with the objects, once identified, have to be classified: positive, if the object is lifted and manipulated; negative, if the object is replaced.

**Figure 17:**
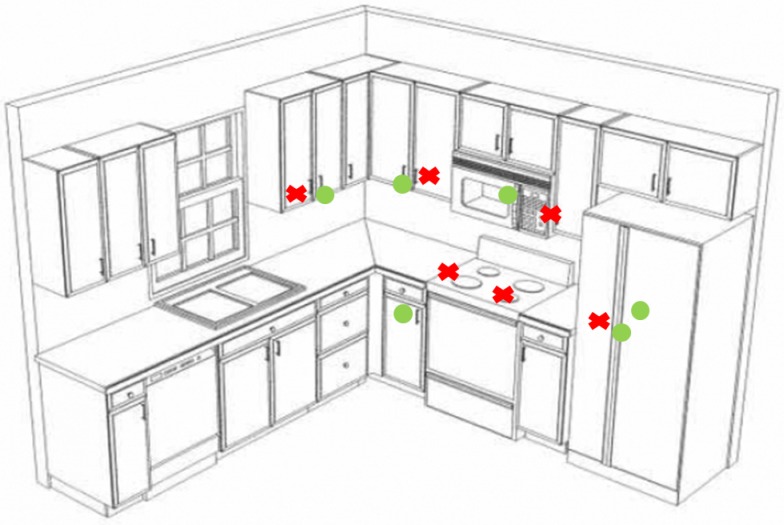
Example of interaction map.

Figure 17 shows an example of a home environment (i.e., a kitchen), where the map of interactions is showed. A positive interaction (circle) means that the object (e.g., a pot or cooking product) is taken from the cupboard and used. Negative interaction (crosses) means that the object was picked up and stored immediately without using it. Memorizing and then processing this information it is possible to establish which is the normal behavior of the user, identifying anomalous situations that can activate alarms (for example a wrong feeding). The experimental phase has demonstrated that the system is able to monitor user behaviors, interactions with other subjects and in particular interactions with the objects. Moreover, users can develop actions using vocal commands and in case of alarm, a series of agreed words can activate a VOIP communication.

### 6.4. Smart Shoes with Localization Functions and Analysis of Motor Activities

In recent years, thanks to the enhance of efficient systems to energy recovery, and the continuous research of systems and materials can capture and transform different type of energy into electrical form, a high number of wearable electronic devices has been developed [50,51]. Mainly, they have been used for monitoring human activity, allowing not only to examine the state of body and the health of people, but also to extract information concerning several fields of application: human motion [52], geolocalization [53], security and many others [54]. Taking into account wearable electronics devices, the most efficient systems for energy capturing are those use Energy Harvesting systems [55] inserted into the shoes. These systems are installed into the soles where, during the walking and the running, the force is exerted. Using piezoelectric elements and electromagnetic induction systems, this force allows to recover high quantity of electrical energy useful for sensor supply and complex monitoring systems [56,57]. For that, it is necessary to analyse the pressure distribution along the sole of the shoes during the walking and/or the running [58], in order to optimize the positioning of the elements useful for the energy recovering. For further details see [59].

**Figure 18:**
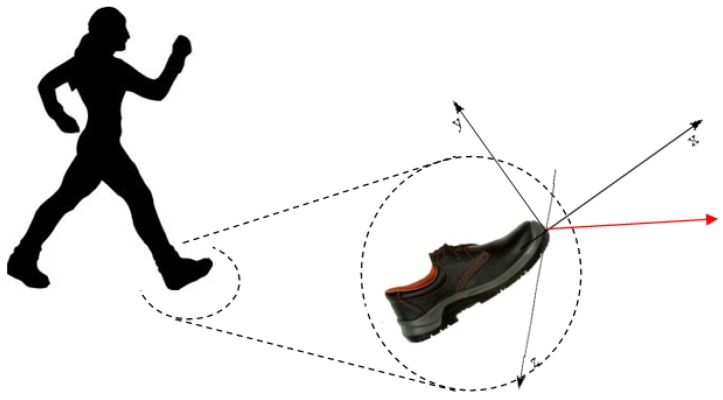
Representation of three directions during the walking.

In this project, the main hypothesis of using smart shoes is related to data acquisition (data logging) for which the system, after it was shod, is able to acquire data and to storage them inside the micro-controller memory, basing on its dimensions. The availability of these data allows to calculate distances covered during the period of use of shoes. Through a radio module, at the end of the period of using, data are downloaded to an information system that effects data post-processing and storage on a database. The ST Microelectronics MEMS sensor is used to give in output 100 or 400 samples per second in the 3 space directions as Figure 18 shows, and the wake-up duration counted after exceeding a pre-set threshold of acceleration.

At the beginning of each step performed with the footwear, the acceleration threshold (suitably calibrated) is exceeded and starts the acquisition of acceleration samples and the counting of the wake-up timer that calculates the duration of the step. The samples acquired, in a variable number according to the length of the step, are filtered or averaged to obtain the average acceleration, then the resulting vector from the three space directions is calculated. The space covered is determined by the following simple equation: $space=a\times t^{2}$ (where a is the acceleration and *t* the time). The used devices have a memory of 32 Kbyte and so, the maximum availability of data storage is the length of 27,648 steps and summing them the distance covered during the day by a person wearing the footwear can be obtained. It is possible to formalize a cycle of typical using of the system:

- start of the day: activation of the system;
- period of actual use: data acquisition;
- end of day: data download and deactivation of the system.

**Figure 19:**
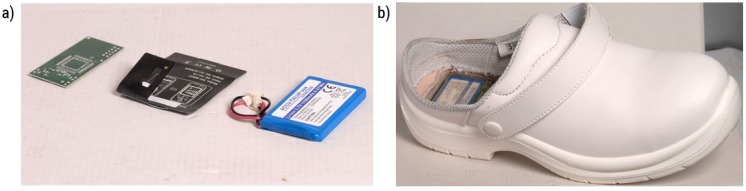
Components (**a**) inserted in the heel and the shoe prototype (**b**).

The system to be inserted in the heel of shoes is made in Figure 19 from left to right:

- Electronic board;
- system of recharge at induction;
- Battery;

In order to insert previously listed elements in the heel of the shoe, a container has been projected and realized using a 3d printer for rapid prototyping. The size of the SO inserted into a compartment in the heel of the shoe are standard 5 × 6 cm. For mass production is provided an easy mounting of the device, pre-injection, in the innersole of the upper. Once injected the material of the fund it will be inextricably incorporated to the sole of the shoe. The experimentation of the smart shoes can be divided into two main parts: the system analyses data deriving from smart shoes, and the interface towards external world in general and to other systems that make HDomo 2.0 in particular. Shoes are in a prototype phase so the model has been chosen to be worn inside the home and suitable for an elderly person. In order to test the system, router have been positioned in different environments of test and the environments have been defined assigning them typical names of a house (kitchen, home bathroom and so on). An operator wears smart shoes and moves in the home environment, and the software recognizes the exact position of the operator. Moreover, the operator tests possible states provided by the firmware:

- falling;
- standing idle;
- extended idle;
- extended active;
- standing active;
- slow/dragging walking;
- normal walking;
- fast walking;
- running;
- anomalous situation;

The integration with the domotic system of building automation of HDomo project is possible thanks to the interaction with Planet Automation (PA) of Proietti Planet. In case of alarm, for example following a fall, or an extended inactive state, the software system sends in dual mode a signal:

- sending an e-mail: this type of alarm is not immediate even if it can be useful to contemporary advice more people also distant from the assisted.
- signal to Planet Automation system: in this case the signal is timely and it is possible to immediately intervene.

### 6.5. Smart TV and App for Devices Management and the Information

A smart TV is an application program that runs on an application engine inside digital television connected to Internet. It is possible to develop novel applications that integrates and extends the offering for users that use smart TV. The use of commercial product offers two main advantages:

- To reduce time to market and the scalability of products using focusing on the application development rather than the platform.
- To reduce the distribution complexity and the application management focusing on the resolution of possible application problems rather than the software distribution.

Our project choice is oriented on the suite of “Samsung Smart TV” products, to have:

- Distribution channel of native software (also eventual upgrades);
- Production Line widely distributed and available on the market;
- Wide range of products that guarantee the functioning of applications on a range of different (but compatible) installations.

The Samsung Smart TV service offers the ability to extend functionalities of the TV by applying a wide range of web functionalities that can be customized by users. With the installation of Samsung Smart TV, users can directly run Internet applications on the TV screen. An application is special type of Web page that is implemented on web browser and is executed on television. So, the system uses a browser integrated on the smart tv that supports following standard: web standard (HTML4.01, XHTML1.0, XML1.0, Markup language specifications HTTP1.0/1.1, CSS1, CSS2, CSS3, CSS TV Profile 1.0, DOM1, DOM2, JavaScript 1.8), Supported image formats (BMP, JPEG, PNG, GIF) and Supported Shockwave Flash formats (Flash10.1/ActionScript2.0/3.0 AIR for TV 2.5.1). In this context, the Smart TV application allows interfacing with external systems, from suppliers of multimedia contents to building automation systems, through HTTP protocol and JSON calls. For the first, Backoffice Manager designed by Websolute, CMS – Content Management System has been used to manage multimedia contents (news, images, videos, and so on) to be reproduced on demand on smart TV. For the integration with domotic systems of building automation an integration with Planet Smart Automation has been realized (PA of Proietti Planet). Among the main characteristics of the system there are:

- **Hardware Abstraction Layer**: it allows to interact with heterogeneous domotic devices through standard API, independent from the technology and the underlying hardware platform;
- **Gateway software** for integration and interoperability among non compatible hardware. It allows to communicate among them hardware belonging to different communication technologies and to establish cause and effect relations. It is possible to program rules under which an event generated by a device of technology A can trigger a command on a device of technology B.
- **Virtual devices**: PA can integrate external and of third parts services as devices, defined virtual devices. For example, it is possible to integrate a social network as Facebook or Twitter or interfacing with a meteo service.
- **Symbolic devices**: it is possible to associate passive devices to smart domotic devices in order to obtain a transparent and intuitive management for the user. For example it is possible to manage a traditional lamp through a domotic actuator as a device endowed of their own domotic functionalities;
- **Combines interoperability with versatility and efficiency of communication**: PA is implemented as Windows Service able to interface with the external world through API based on Windows Communication Foundation technology. Client can choose to communicate with PA through endpoint based on Web service or to maximize the performances through binary binding on TCP/IP channel.
- **API available in two modes**: synchronous and asynchronous. PA client can choose whether to communicate with the service in synchronous or asynchronous mode. It is also possible to use contemporary two modes in the same client. The asynchronous mode provides an efficient communication to events: when something occurs, server send a notify in push mode.
- **Device hot-plug**: PA is prepared for warm start of new devices within the plant configuration. It is not necessary to stop or reset the system so that new devices will integrate and start working.

The user interface of the application is designed to be usable by an elderly person. It is assumed that a caregiver helps the elderly person in the preliminary learning phase of the application.

### 6.6. Audio Analysis System for Speech Recognition and Novelty Detection

The most versatile and minimally invasive sensor for the AAL is the microphone that allows to capture a continuous signal from the home. Examples of audio framework related to the automatic recognition of controls and emergency calls within a domestic environment are processed in the following articles [60,61]. A microphone continuously monitors acoustic sounds present in a dwelling identifying among them the words through a Voice Activity Detector (VAD). Concerning the VADs, many algorithms have been developed all based on the energy of signal recognized as word: assuming that at the beginning of the audio there is no words pronounced, this energy level is recognized as threshold and only whenever this is exceeded, it is possible to identify a word and then pulling it out from the background noise [62]. Voip call begins once pronounced by the user a sequence of words recognized by the system as an emergency call (for example, “Help help help”), allowing the user to launch a communication with a family member or a service center to request help. Other vocal segments recorded are delivered to an automatic speech recognition system (ASR) which identifies vocal commands and consequently activates processes that leads to perform actions related to them, such as turning on the lights. The vocal recognition module is based on PocketSphinix [63]. PNCC (Power Normalized Cepstral Coefficients ) algorithm is developed in C language and integrated in PocketSphinix. The acoustic model and approach developed to exclude words from the grammar is described in [60]. Using algorithms in [64] an improvement of performance can be obtained by integrating a recognition classifier tone of voice effort.

### 6.7. Gesture Analysis Systems

This part of the project has the task to study and realize a system of user detection that is also able to recognize the user movements in order to provide a natural interface that can be used without the use of external devices and that with other HDOMO 2.0 devices makes easy user interaction also for users with injury or disabilities. First of all, the detection system is described then the development environment chosen and finally the description of the developments. To allow the user to configure commands for any gesture was also realized an apposite interface. So that a user with a digital literacy enough for navigate websites can also configure their own.

After the study on the state of the art of sensors and after having examined the systems considered most interesting (area of operation very restricted and need to make too precise movements for people with limited mobility), based on the needs of the project as sensor has chosen to use a structured light system provided by PrimeSense (Kinect, Asus Xtion, PrimeSense Carmine), in particular the version of Microsoft for the following reason:

- The sensor does not require any peripheral, even passive to wear by the user;
- The sensor allows to have a sufficiently large ($57^{\circ }$ in horizontal) and until to 8 m of distance;
- The sensor is easily available, economic and easy to install;
- There are alternatives from other manufacturers equal and compatible;
- The system is supported by libraries and drivers from Microsoft and third parties.
- The sensor is able to contemporary detect gestures of one or more users;
- The sensor in indoor environment can ignore the quality of illumination and it has not problems of chromaticity with clothes of user. Even if it has the only restriction that the zone is not struck by sunlight and that possible obstacles do not prevent, however, the direct and complete view of the user.

The sensors are compatible with different environment of development and OS. The choice was as development environment Ubuntu 12.04 LTS Linux distribution, Java 7.0 as language, OPEN NI AND OpenCV as driver and low-level library, using IDE Eclipse as development environment. These choices are motivated by the need to produce a portable and open system. Then:

- OS is open source;
- The language is extremely portable;
- drivers and libraries are open source and compatible with numerous OSs;
- the development environment is also known and portable on different OS.

A protocol of communication between sensor and user has been defined. It is based on TCP-IP standard communication with messaging in XML. To allow the user to configure the commands underlying each gesture, a special web interface has been created. Below is a list of available movements that can be associated with an action (e.g., switching lights on or off, raising or lowering the cooker hob, opening kitchen cabinets, turning on smart TV, etc.):

- left-hand-hand-push: press with the left hand.
- Left-hand-swipe-down: Move the left hand from top to bottom.
- Left-hand-swipe-left: Move the left hand from right to left.
- Left-hand-swipe-right: Move the left hand from left to right.
- Left-hand-swipe-up: Move the left hand from bottom to top.
- Right-hand-push: press with the right hand.
- Right-hand-swipe-down: Move the right hand from top to bottom.
- Right-hand-swipe-left: move the right hand from right to left.
- Right-hand-swipe-right: Move the right hand from left to right.
- Right-hand-swipe-up: Move the right hand upwards from below.
- Zoom-in: Extend both hands horizontally.
- Zoom-out: narrow both hands horizontally.

### 6.8. Non-Invasive Analyser of Respiratory Rate

**Figure 20:**
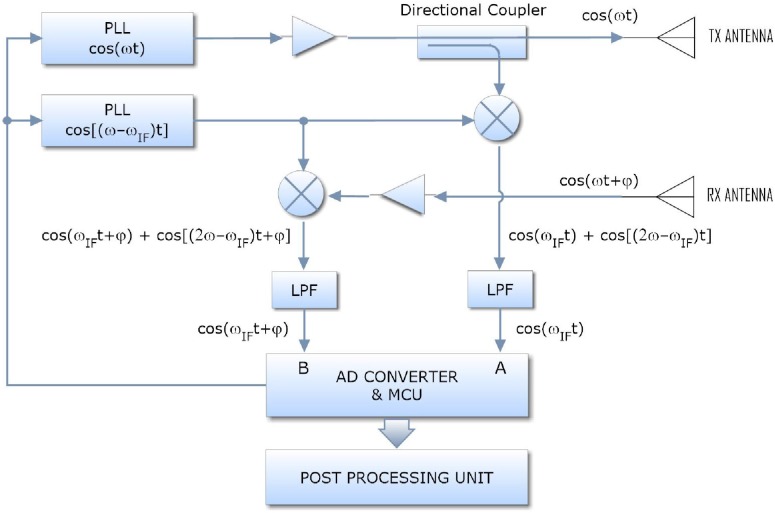
Block diagram of the prototype RX/TX unit for HDomo 2.0 Project.

The respiration rate is one of the physiological signals (also known as vital signs) that are generally monitored in patient observation because it is considered an important predictive parameter for many pathologies. In general, the traditional procedures to gather vital signs from human body require the use of one or more transducers, electrodes, cables to be connected to the patient. A possible approach to the problem is represented by the use of electromagnetic (EM) technology [65,66] , which is able to ensure: non-contact measurements at significant distances (a few meters), and most importantly, it can work also through tissues (i.e., bed sheet, blankets, clothes), because common fabrics are generally transparent to EM waves. Moreover, an EM system may be installed inside a home without compromising end user privacy and may be hidden into the wall or ceiling so not having a significant impact on the appearance of the rooms where it will be installed. The final block diagram of the realized device is shown in Figure 20. The solution with two antennas instead of a single one has been preferred for cost reasons and reduction of circuit complexity: The proposed solution on other hand has the only drawback that the system is more bulky and it must be taken care to decouple the antennas to prevent that the transmitted signal enters directly into the RX channel.

The core of operations is the microcontroller. The device chosen is an ARM-based Cortex-M4 processor with the following features:

- Single-cycle multiplication and HW division;
- 1.25 Dhrystone MIPS/MHz and DSP instructions;
- Max operating frequency is 72 MHz (but in the design it is working at 70 MHz);
- 256 Kbytes of flash memory and 48 Kbytes of RAM;
- 4 ADC’s that can operate up to 5 Msps
- Interfaces: CAN, IC, SPI, USART/UART, USB, Infrared.

**Figure 21:**
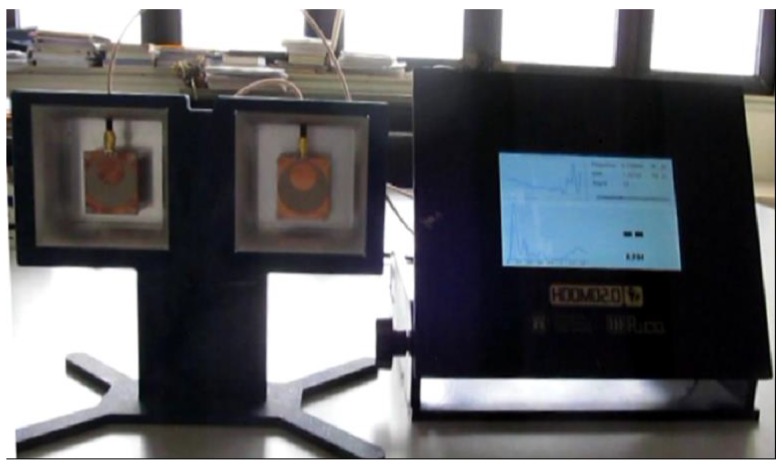
The stand-alone realtime device working at a single frequency.

Two external PLLs are used to set the transmitted frequency and the demodulating frequency. These devices allow the generation of RF frequencies up to 6.39 GHz with a resolution of 38 Hz, therefore they can be used to generate the desired frequency sweeps with a significant granularity. Finally, except for a factor of proportionality, it is possible to obtain an estimation of the S11 parameter. Since the microcontroller has the capability to sample at Fs = 5 Msps, it is then possible to obtain 10 samples per period for each wave at the intermediate frequency (FIF = 500 kHz). The emitted power of the standalone prototype is 11 dBm and it allows to determine the breathing frequency up to 4 m. The final system has involved, as the post-processing unit, the use of an embedded Linux system for the transform computation and data display. The system used for high-level computation is a commercial U-Mobo board, which runs Ubuntu 12.04.3 LTS. This solution has been chosen because of the reduced size of the board and high computation potentialities (up to 24 GFLOPS). Figure 21 shows the final device boxed: on the left are visible the TX/RX antennas, while on the right the display (with touch screen) of the Linux system.

**Figure 22:**
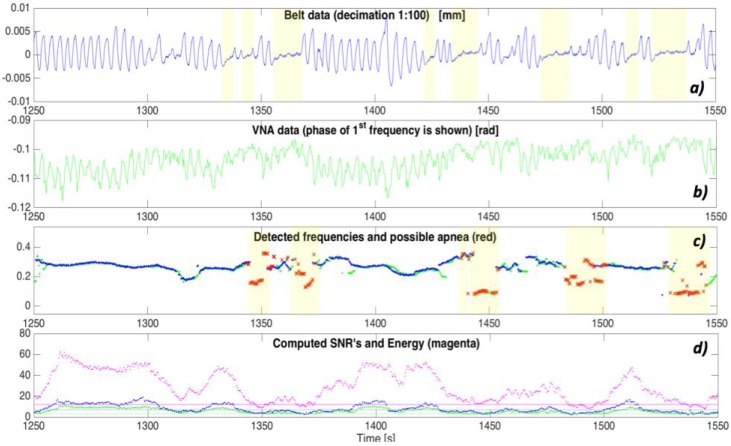
Exctract of a 50 min sleep monitoring: belt data (**a**); EM data (**b**); comparison of detected breathing frequencies (**c**) for 5 min of sleep (blue and green points are the frequency peaks of Fourier transforms of respectively belt and EM data); computed SNR and energy (**d**).

With this device it is possible to detect changes in the respiratory rate so alarms can be triggered when the breath drops or drops or rises above a certain threshold. It is also able to detect apnea events, a serious problem that affects adults and infants and can lead to irreversible brain damage and even to death. Testing was performed to verify the recognition quality by comparing the respiratory rate with the respiration belt [67] . Figure 22 shows an extract (5 min) of the 50-min day trace. In details, Figure 22a,b respectively report the belt time Signal and the EM data from the VNA, while Figure apnea-50 min) shows the comparison of the frequencies detected with both systems. The blue (belt Detection) and the green (EM detection) points are almost everywhere overlapped except when apnea episodes occur. The algorithm also evaluates the SNR and the energy associated to the EM signal (magenta line in Figure 22d) and compare them with two corresponding thresholds. As result, if both SNR and signals energies are below the threshold, it marks the values obtained with the EM system with red crosses, indicating the possible presence of apnea, otherwise no red crosses appear.

### 6.9. System for Easy Access at Home in an Emergency Situation

The communication system consists of three major devices:

- Phone Device: offers simplified call to relatives, caregivers, hospital etc. It provides automatic response mechanism with hands-free activation for preset numbers (useful in the event that the person has an illness).
- GSM and Control Electrical Network Device: The GSM device monitors the status of the electrical network and, in case of interruption, send an SMS to predefined numbers, also provides a simulated telephone line to which any telephone terminal can be connected to make or receive calls and a relay controlled by specific SMS messages. The device is also arranged to send information of any type directly to a dedicated web server (cloud).
- Video entry device with smart reader: this device allows the access control house via RFID proximity reader (TAG Reading compatible EM4002 at a distance of 3–5 cm—Operating frequency 125 KHz). The smart object can be connected directly to the telephone line or extension, the GSM device, and when a call coming, can answer and open the doors through specific codes entered from the keyboard. It is also able to receive the signal of presence or absence of an “alarm” directly from the local gateway, accordingly enables or disables a series of RFID cards and access codes “passe-partout” to allow access to rescue personnel.

**Figure 23:**
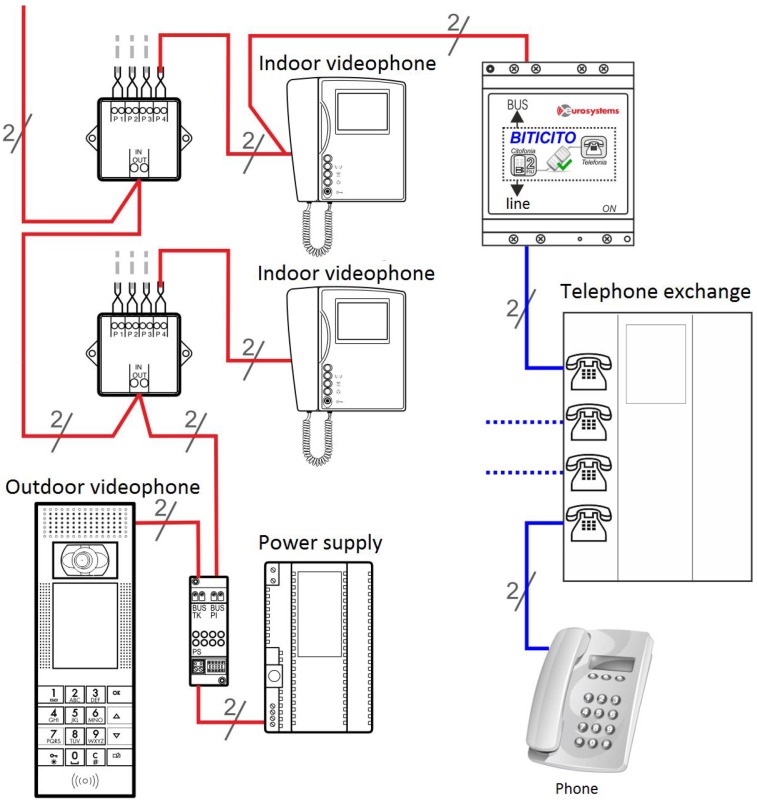
Description of the videophone system.

Figure 23 shows the connection of video door entry system. The outdoor device is connected with the indoor device using a GSM module. The BITICITO module allows you to connect the videophone to external phones/smartphones.

## 7. HBA and Machine Learning

Data collected in the AAlaaS system are in real time processed for alarms management. The possibility to extract information in the form of raw data resulting from monitoring, makes the monitoring software used in HDOMO an extremely powerful tool for carrying out analysis. The main purpose was to be able to learn from correct processes and classify, and then manage and detect any abnormal behavior which may be attributable to alarm situations inside the home. This system was implemented in the WEKA framework for classification and data mining. In the experimental phase the most relevant items for the analysis of the system are considered:

- Breath analysis: You can create alerts based on the change in respiratory rate. Many studies [68,69,70] suggest that this analysis allows to detect many diseases.
- Interaction with the kitchen counters and appliances: You can study the behavior of the individual within the home automation kitchen in order to identify the frequency of daily meals, the hours in which they are consumed etc.
- Floor: you can control the behavior of an elder in the house watching the paths that performs daily and generate alarms if emerging attitudes and abnormal movements.

For each system was done an initial training using a manually labelled dataset that describes a normal behavior. In general, most of items give a binary output (ON-OFF) and to each item it is linked a timestamp describing the instant in which the data has been received. Concerning the floor, it is not possible to provide a depth experimental analysis since the prototype consists of only three tiles. Possible future developments are very interesting because it will be possible to control the behavior of the elderly inside his home by observing the paths that he daily performs and generating alarms if there are attitudes and unusual displacements that indicate any loss of memory. Other information concerns the walk of the elderly, in fact the system could signal an anomalous situation if the average time between a step and the other is different from a normal value.

### 7.1. Classification Model

In this section, firstly, we provide a brief overview of the used machine learning method. The method is very well known in the literature and the purpose of introducing machine learning in HDOMO is mainly to prove that the set of Smart Sensors here proposed and integrated on the same architecture can benefit from machine learning approaches to perform an intelligent integration of data coming from every sensor in behavior detection. Then for the solution to this problem a cross-validation (CV) procedure is applied. The approach chose for this problem is k-fold CV, so the training set is split into k smaller sets.

#### 7.1.1. Support Vector Machine (SVM)

Support Vector Machine (SVM) [71,72] is a constructive learning algorithm, that belongs to a family of generalized linear binary classifiers. It maps an input feature vector into a higher dimensional space and find a hyperplane that separates samples into two classes. In this way that the margin between the closest samples in each class is maximized. In high dimensional data classification, it has shown great promise [73] and it has been successfully employed to AAL/biomedical data mining [74].

#### 7.1.2. Evaluation

A 10-fold cross-validation has been applied to ensure the robustness of performance estimate [75]. The performance of different classifiers and feature sets was evaluated in terms of precision, recall and F1-score (F1) using weighted macro-averaging over 10 folds.

To evaluate the performance of the algorithms the following metrics are employed:

- *Accuracy*: approximates the effectiveness of the algorithm by showing the probability of the true value of the class label [76]:
  (1) $$Accuracy=\frac{t_{p}+f_{n}}{t_{p}+t_{n}+f_{p}+f_{n}}$$
  where $t_{p}$ is the number of true positives and $f_{n}$ the number of false negatives.
- *F1-score*: is a measure of a test’s accuracy.
  (2) $$F1-score=\frac{(\beta^{2}+1)\times precision\times recall}{\beta^{2}\times precision+recall}$$
  The F1-score is evenly balanced when β=1. It favours precision when β>1, and recall otherwise. The F1-score can be interpreted as a weighted average of the precision and recall.
- *Recall*: is a function of its correctly classified examples (true positives) and its misclassified examples (false negatives).
  (3) $$recall=\frac{t_{p}}{t_{p}+f_{n}}$$
- *Precision*: is a function of true positives and examples misclassified as positives (false positives).
  (4) $$precision=\frac{t_{p}}{t_{p}+f_{p}}$$
- *Support*: is the number of occurrences of each class in ground truth (correct) target values.

The information about actual and predicted classifications done by a oocytes classification system is depicted by confusion matrix [77]. Confusion matrix is a specific table layout that allows visualization of the performance of an algorithm, where each column of the matrix represents the instances in a predicted class and each row represents the instances in an actual class. In the result section, we will report the accuracy of every sub-system and of the integrated system based on the previously described methods.

## 8. Test Case and HDOMO Demo Environment

### 8.1. Test Case: The HDOMO Demo Environment

The aim of this section is to describe a possible HDOMO scenario that allows to have an overview on the interaction among different SO and the consequent chain of events that is generated when a specific alarm occurs. This case study also aims to show how the entire system is able to react to an alarm event and the ability of the gateway of the house to coordinate in parallel and automatically many sequences of actions designed to secure the elderly person. Finally, this test made it possible to prove the robustness of the system in case of possible connectivity problems. We assume that an elderly person lives alone inside his domotic home. The elderly falls on the floor and is unable to get up after the fall. The floor through its sensors detects the person falls on the ground and then sends an alarm signal to the central gateway, the device that acts as interface towards the outside home. Then the Gateway performs three tasks:

1. Send a message of alarm to the automatic switchboard capable of starting in a completely automatic way a telephone call to the health professional. Through a virtual assistant the operator knows the type of accident occurred. The virtual assistant remains pending to receive a voice notification of receipt of the message. The switchboard addition to the call is able to send SMS useful to alert relatives of the elderly.
2. Send a message of alarm to the web cloud platform for remote management. Automatically also the web platform will notify to the automated switchboard the fall to obtain an alert mechanism structured on several levels and therefore more robust to possible network problems. Cloud service allows to maintain a history of all activities conducted by SOs and then it is possible to also verify previously calls and to control the timing of rescue.
3. Send a message of alarm to the videophone of the dwelling that automatically enables an array of RFID tags predefined for this type of alarm. The healthcare provider that has the RFID will enter in the home of the assisted only after to have inserted his pass-partout code.

When the operator receives the call alert, can try to call the patient equipped with a GSM unit can automatically respond to the phone and set the speakerphone. In this way, the operator can hear what is happening inside the house (around the phone) and can groped to reassure the patient.

**Figure 24:**
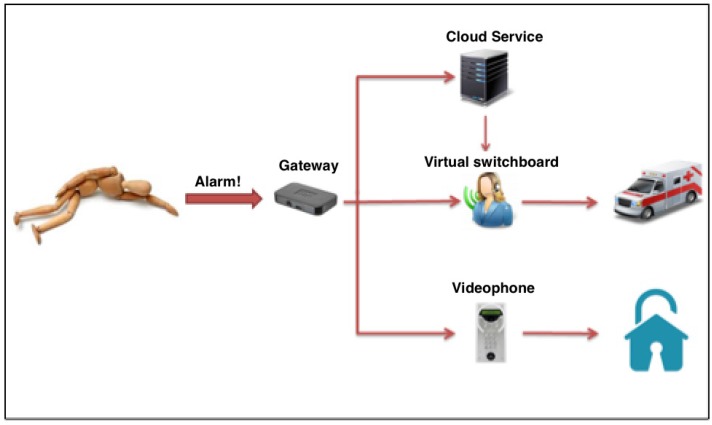
Example of possible HDOMO scenario.

Figure 24 shows a scheme of the sequences previously described. Departing from the accidental occurrence until help arrives.

### 8.2. HDOMO Demo Environment

The individual SO communicate low-level data (for example temperature or localization of the person in the environment) towards the domestic gateway (HDG = gateway + Planet Automation) without a standard of reference at this level of communication. Each producer of SO has access to the SW layer of HDG (embedded platform that offers only a common monitoring and diagnosis layer) and it is responsible for developing the low-level connection module. The standardization phase occurs between HDG and a high-level layer that it is responsible to analyze data coming from sensors. This layer is realized according to the Cloud computing layer and offers AAL as a Service (AALaaS). For high level interactions between HDG and AALaaS, web services have been defined both for the exchange of different typologies of data coming from different SO and verifying the configurations. Inside the Cloud environment occurs high-level processing related to the classification of users behaviour based on actions (abstraction of measures of different SO and their sensory fusion). This environment is also responsible of alarms managing on different emergency levels.

The test environment was set up with two rooms of a HDOMO test home: entrance and kitchen. In the environment, there are all the HDOMO SOs and two gateways.

**Figure 25:**
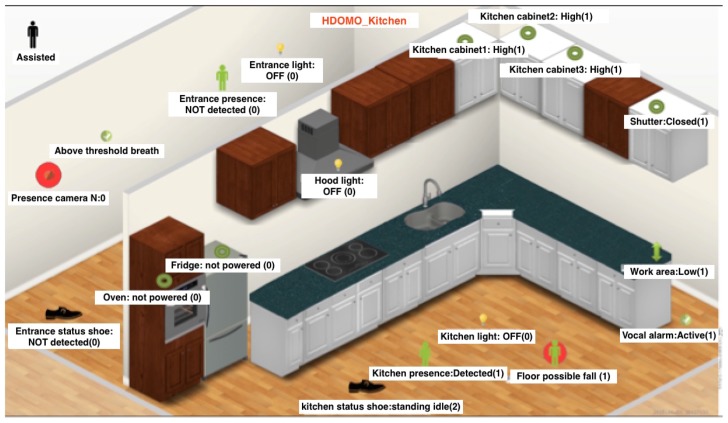
Example of a HDOMO environment.

The experimental set-up is structured in an interactive path able to involve the user the benefits of introducing systems developed by HDOMO. The main purpose of creating this environment was to be able to carry out the tests presented in the next section using a real environment and collecting real data. HDOMO test case is currently an open lab in Italy that helps SMEs to develop new products for AAL. In particular, the user can test different functionalities in an environment endowed by several SOs. As for example:

- Use an interphone able to prove an easy access to the house in case of alarm, through a slim distribution of virtual keys (numeric codes or QR);
- Interact with the environment through interaction systems made easier for the weak user: a remote control for interacting with the environment, a voice recognition system in Italian and in the local dialect for access to the management of the lights and the handling of kitchen cupboards;
- Use gestures to interact with other SOs (for example, to turn on the lights the cooker hood or open a door);
- Collect interactions data between user and kitchen to understand behaviors related to feeding habits and the risks of the kitchen (as in Figure 25).
- Localize people through more alternative or complementary systems: an innovative camera battery, a bracelet or a remote control locatable in the home, a smart floor and so on;
- Obtain in real time information on falls both through vision that through interactions with the smart floor;
- Analyze breath in non-invasive way and collect data in the cloud area of HDOMO management to handle alarms;
- Use an innovative smart shoe able to localize, identify falls, estimate the daily moving of the user, using a self-recharge system;
- Having an innovative telephonic console able to intelligently manage incoming calls and outgoing.

All data generated by aforementioned systems are collected and managed by internal gateways that communicate to a cloud infrastructure all information necessary to classify behaviours and handle alarms. All user interfaces are usable through three platforms in HDOMO project developed: a Web portal that provides all the advanced features, two applications (for tablet and smart TV) with sub functions useful for the user or the assistant.

## 9. Reliability Test of the Data Acquisition and Analysis System

The demonstrative environment has been used to conduct simulated tests to verify the reliability of the network infrastructure and the data acquisition system in receiving events from smart objects. The controlled environment test was carried out on 6 volunteer researchers who alternated in the execution of predefined tasks (the actions to be performed were specified but not the order in which they should be performed). At this stage we did not consider it necessary to test with elderly people because the main objective was to have a high number of activated alarms with an annotated dataset shared among SMEs working on the different SOs development. For 7 days different types of alarm were artificially created and detected by smart objects, verifying through visual inspection whether the alarm was actually detected by the system as well. The purpose of this test was to analyze system reliability by distinguishing correctly detected alarms, false positives and false negatives, applying a collaborative approach between the SOs. It is important to note that the alarms/events detected by smart objects are read on Zabbix interface and not directly from the smart object. Table 1 shows the 7 smart objects selected to detect alarm situations and the type of events they are able to detect (e.g., shoe can detect walking, running, falling, standing idle, etc). Table 2 shows the behavior of SOs in recognizing 6 types of alarms. The “# Events” column represents the number of events, established in advance, that will be executed by the volunteer in a random sequence (for example, for the Fall alarm, 500 events have been generated that can be recognized by the Smart Floor). The “% Event Accuracy” column represents the number of events correctly detected by the individual smart object compared to the number of events actually occurred and confirmed by a visual inspection (75% of events detected correctly by the smart floor). The “# Alarms” column represents the number of a priori alarm situations that the volunteer will perform within the entire sequence of events (100 fall alarm situations). The column “% Alarm Accuracy” represents the number of alarms correctly generated by the collaboration of all smart objects selected to recognize that particular alarm (94%), based on the ML approaches described before. This type of information cross-referencing makes it possible to obtain a higher degree of accuracy than a single smart object, reducing the number of false positives and false negatives. In each alarm situation, weights were assigned to each smart object based on its ability to recognize that particular alarm.

Table 2 describes, for each SO, the number and percentage of the generated alarms (e.g., the alarm triggered by alarms when the breath frequency goes above or below the threshold) together with the overall alarm accuracy (measured in percentage) with respect to the ground truth observed and annotated during the test week. The overall accuracy is really high and the data fusion and concurrent behaviour understanding. We also tested the user interface in terms of usability and acceptance of the system with a total of 100 stakeholder. In general, more than the 90% of the total caregiver that was asked to give a score between 1 and 10 for every alarm kind in term of acceptability and usability reached a quality level major than 9. Results demonstrated the feasibility of the system and its accuracy showing that the correct approach and the main results of HDOMO are related to the correct mix of different SOs that are low cost, easy to install and manage and interoperable. At the current stage HDOMO is the largest set of SOs in Italy and Europe able to interoperate on a common architecture.

## 10. Conclusions

The paper has described the HDOMO project, which involved 16 SMEs and 2 research institutes in the development of a complex system that includes several SOs. The project proposes an innovative idea of an interoperable embedded intelligent system where different low cost intelligent sensors can analyse human behaviours to promptly respond to the needs of users and to obtain statistical data useful for future processing. The main target was the development and the implementation of a platform for AAL applications that starts from the integration of existing platforms and extends the concept of SO interoperability. The project was financed by Marche Region and faces the challenge of developing different layers of abstraction using different SOs to obtain a very accurate alarm system for AAL.

A desirable feature in home automation concerns the so-called flexibility of interfaces, or the ability to use different devices to give commands to the automated home. In our case this is possible because the command is no longer tied to the physical cable, but is transmitted with a message and information so that different devices can activate in parallel the same function. Flexibility of interfaces means choosing, regardless of the configuration and functions of the home automation system, the control device that best suits the needs of the user. In the case of people with disabilities, it means to use the commands that can make the most of the remaining abilities, using all those dedicated devices designed to adapt to different situations of disability, e.g., for those who can only move his arms, for those with only a residual movement, for those who can only use the voice or for people who can interact through the computer. To send commands, many of these devices use dedicated infra-red transmission, as most of the remote controls present in the home, for which it is sufficient to provide an infra-red receiver to integrate them in the system, without requiring special or particularly evident devices. This ensures that an automated home, although designed with special solutions for people with disabilities, may have the image of normality necessary to maintain the appearance of a familiar and welcoming environment.

The data management system has been tested by simulating a house equipped with all the SOs HDOMO defined in the project (about 30). The aim was to check the behaviour of the system on speed, that is, when all SOs simultaneously send information on their status. Several tests were carried out also modifying the frequency with which the system interrogates the various objects (for example every 5 s). The tests have highlighted problems relating to the management and storage of data, because the system is arranged to simultaneously monitor hundreds of items without slowing down. In this phase, two aspects were considered.

- First, the implementation of an experimental classification system that retrieves real time data provided by items and, after training, can generate alarms when necessary. This system ensures an automatic management of alarms and allows to detect relevant information that can not be inferred from data visualization.
- Second, the performance analysis of a system connected to hundreds of domotic homes, which therefore must be able to simultaneously handle a greater number of SOs. Considering, for example, 50 items, about 10 Gb will be produced in one year, so that the use of scalable architectures for storage, analysis, and sharing of large amounts of data is necessary.

## Figures and Tables

**Table 1:** Type of event detected by SOs.

Smart Object	Event Type
Smart Floor	Standing idle, slow/dragging walking, normal walking, falling
Smart Camera	Location tracking, falling, Kitchen interaction
Localization System	Location tracting
Smart Shoes	Standing idle, extended idle, extended active,
	standing active, slow/dragging walking, normal walking, fast walking, running,
	anomalous situation, falling
EM Respiratory Rate	Normal breath, apnea, breath below threshold, breath above threshold
Smart TV and Mobile App	Interactions with the device, Location Tracking
Speech and gesture Recognition	Help request, Interaction with the device

**Table 2:** HDOMO Testing scenario and Accuracy Results.

Behaviours	Alarm Type	Smart Object	# Event	% Event Accuracy	#Alarms	% Alarm Accuracy
Movement Activities	Fall Detected	Smart Floor	500	75	100	94
		Smart Camera	300	80		
		Smart Shoes	800	82		
		Localization System	200	90		
	Dragging Feet	Smart Floor	400	61	50	87
		Smart Camera	200	65		
		Smart Shoes	600	79		
		Localization System	100	90		
	Standing Inactive	Smart Floor	400	80	50	96
		Smart Camera	200	85		
		Smart Shoes	600	86		
		Localization System	100	90		
Respiratory Activities	Night Apnea	EM Respiratory Rates	3000	98	150	100
		Smart Camera	50	70		
Daily Activities	Help Request	Speech and gesture Recognition	400	85	100	100
		Localization System	200	92		
		Smart TV and Mobile App	300	95		
Feeding Activities	Incorrect Feeding	Smart Camera	300	90	30	92
		Speech and gesture Recognition	200	85		
		Locallization System	50	89		
